# Accumbens D2-MSN hyperactivity drives antipsychotic-induced behavioral supersensitivity

**DOI:** 10.1038/s41380-021-01235-6

**Published:** 2021-08-04

**Authors:** Anna Kruyer, Jeffrey Parrilla-Carrero, Courtney Powell, Lasse Brandt, Stefan Gutwinski, Ariana Angelis, Reda M. Chalhoub, Thomas C. Jhou, Peter W. Kalivas, Davide Amato

**Affiliations:** 1 Department of Neuroscience, Medical University of South Carolina, Charleston, SC USA; 2 Department of Psychiatry and Psychotherapy, Charité – Universitätsmedizin Berlin, Berlin, Germany

**Keywords:** Neuroscience, Psychiatric disorders

## Abstract

Antipsychotic-induced dopamine supersensitivity, or behavioral supersensitivity, is a problematic consequence of long-term antipsychotic treatment characterized by the emergence of motor abnormalities, refractory symptoms, and rebound psychosis. The underlying mechanisms are unclear and no approaches exist to prevent or reverse these unwanted effects of antipsychotic treatment. Here we demonstrate that behavioral supersensitivity stems from long-lasting pre, post and perisynaptic plasticity, including insertion of Ca^2+^-permeable AMPA receptors and loss of D2 receptor-dependent inhibitory postsynaptic currents (IPSCs) in D2 receptor-expressing medium spiny neurons (D2-MSNs) in the nucleus accumbens core (NAcore). The resulting hyperexcitability, prominent in a subpopulation of D2-MSNs (21%), caused locomotor sensitization to cocaine and was associated with behavioral endophenotypes of antipsychotic treatment resistance and substance use disorder, including disrupted extinction learning and augmented cue-induced cocaine-seeking behavior. Chemogenetic restoration of IPSCs in D2-MSNs in the NAcore was sufficient to prevent antipsychotic-induced supersensitivity, pointing to an entirely novel therapeutic direction for overcoming this condition.

## Introduction

Antipsychotic drugs are widely prescribed to treat psychosis and many other psychiatric and non-psychiatric disorders [1–3]. Although they are an efficacious short-term intervention in most patients [4], chronic antipsychotic treatment is characterized by high relapse rates in patients with psychosis (70–80%) [5, 6]. Around 39% of these cases result from antipsychotic-induced dopamine supersensitivity [5], or simply behavioral supersensitivity, characterized by decreased therapeutic efficacy and emergence of motor side effects in compliant patients [7]. Although onset of behavioral supersensitivity often motivates patients to reduce or abstain from antipsychotic intake [6], discontinuation of antipsychotic treatment worsens symptoms of behavioral supersensitivity by further reducing efficacy of reinstated antipsychotic treatment (i.e., treatment resistance) [8–10], exacerbating the severity of motor side effects such as dyskinesias [7, 11] as well as psychotic symptoms [12, 13], and increasing the likelihood of future relapse [8, 13].

Antipsychotic-induced behavioral supersensitivity is hypothesized to result from enhanced sensitivity to dopamine via increased expression and/or sensitivity of the D2 receptor (D2r) in striatal neurons following long-term receptor blockade with antipsychotics [14, 15]. Despite many literary references to this hypothetical mechanism, some investigations show that the D2r is increased in roughly half of patients (5 out 9 patients, ~55.6%) after antipsychotic discontinuation (i.e., 14 days) and symptoms do not correlate with receptor upregulation [16]. Likewise, the D2r is not altered in most patients following longer periods of treatment discontinuation (4–6 months) [17–19] and relapse of symptoms appears to be temporally unrelated to putative D2r changes [8]. Furthermore, increased D2r expression or function is associated with high antipsychotic doses [20–22], whereas behavioral supersensitivity is associated with the duration of antipsychotic treatment in animal models [22–24]. Finally, behavioral supersensitivity can also occur during ongoing antipsychotic treatment [22, 24] when D2 receptors are significantly blocked [24]. Thus, while the underlying mechanisms are unclear, defining the cause of antipsychotic-induced supersensitivity remains a fundamentally important issue in psychiatry, since it is frequently observed and poses a severe burden on patients.

In this study, we examined the impact of chronic haloperidol treatment and discontinuation on D1- and D2-MSNs in the nucleus accumbens core (NAcore, the ventral extension of the caudate-putamen), a brain structure with high dopamine release capacity [25] involved in neuronal responses to antipsychotics [26] and in cocaine sensitization [27–29], and describe definitive mechanisms of antipsychotic-induced behavioral supersensitivity. Because the behavioral symptoms and neuroadaptations of antipsychotic-induced supersensitivity are more prominent after antipsychotic discontinuation [7–10], we focused on changes occurring during abstinence following chronic haloperidol treatment. We further identified specific behavioral symptoms associated with antipsychotic-induced supersensitivity in rodents, testing for psychomotor responses to cocaine, expression of vacuous chewing movements (VCMs), a surrogate measure of dyskinesia, and measures of both cocaine taking and seeking.

## Methods

Full methodological descriptions can be found in the Supplementary materials and methods.

### Animals

We used male and female transgenic mice for all experiments except for operant behavior, where we used male and female wild-type Long Evans rats. All animals were bred in house and procedures involving the use of animals were conducted in accordance with guidelines established by the National Institutes of Health and approved by the Institutional Animal Care and Use Committee at the Medical University of South Carolina.

### Locomotor sensitization and cross-sensitization

Mice undergoing cross-sensitization were implanted with subcutaneous osmotic infusion pumps (Alzet) delivering clinically relevant doses of haloperidol at 0.5 mg/kg/day for 14 days [24]. Mice were abstinent from haloperidol for an additional 7 days before undergoing locomotor cross-sensitization with cocaine (15 mg/kg, i.p.). Mice undergoing cocaine mono-sensitization received two cocaine injections (15 mg/kg, i.p.) separated by 7 days incubation as described in [30].

### In vivo Ca^2+^ imaging

To study the activity of D1- and D2-MSNs in vivo we used D1- and D2-cre mice as previously described [26, 31] to achieve selective expression of the Ca^2+^-sensing fluorophore GCaMP6f in D1- and D2-MSNs in the NAcore. Ca^2+^ traces from independent neurons were computed and Ca^2+^ events that were ≥6× the median absolute deviation of the input trace data and with a minimum mean decay of 0.1 s were included. Ca^2+^ events were transformed into binary values to obtain the absolute number of spikes per min over a period of 45 min. The absolute number of Ca^2+^ events that passed the sphericity test were categorized to determine distinct cell response patterns compared to baseline using unsupervised K-means clustering for parametric data. The optimal number of clusters was automatically selected using the fit statistic Cubic Cluster Criterion and subsequently categorized empirically as activated, inactivated, or unchanged.

### Western blotting

Brains were extracted from mice and dorsal striatum, ventral striatum, midbrain, and cerebellum tissue extracts were dissected and manually homogenized in ice-cold RIPA buffer with protease and phosphatase inhibitors (Thermo Fisher). Protein content was determined using BCA (Thermo Fisher) and Western blotting was conducted using standard methodology. Blots were imaged using a LI-COR imaging system and the band corresponding to the D2r was identified based on its molecular weight (~50 kDa) and its absence in cerebellum extracts. Images were converted to grayscale and quantified using FIJI.

### Ex vivo electrophysiology

Selective recordings of D2r-IPSCs in the NAcore were conducted following overexpression of the G_i/o_ protein-coupled inwardly rectifying potassium channel (GIRK2) with a tdTomato-reporter in D2-MSNs. Pre and postsynaptic excitatory recordings were made from Drd1a-tdTomato and Drd2a-eGFP expressing MSNs in the NAcore.

### Confocal microscopy

Mice received NAcore injections of AAV5.GFAP.hM3d.mCherry. After extraction, brains were sliced at 100 µm and sections were immunolabeled by incubation in primary and secondary antibodies. Immunolabeled tissue was imaged using an SP5 confocal microscope (Leica) and Z-stacks were deconvolved (Autoquant) and analyzed for fluorescence intensity using Bitplane Imaris. Astroglia were digitally isolated and their co-registration with Synapsin I was normalized to the astroglial volume and to total Synapsin I signal per equivalent stack size to account for changes in astroglial volume and Synapsin I density. GLT-1 expression that co-registered with mCherry was normalized to the astroglial volume. In all cases, data were ultimately normalized to the mean value of the untreated group.

### DREADD inhibition of D2-MSNs

Mice were implanted with bilateral cannulae (Plastics One) above the NAcore along with osmotic pumps for subcutaneous haloperidol infusion. AAV2.FLEX.hM4Di.mCherry (Addgene) or sterile saline was delivered 1.4 mm beyond the tip of the guide cannulae and virus incubation occurred during 14 days of haloperidol treatment and 7 days of abstinence. Clozapine N-oxide (CNO, 1 mM, 0.3 μL, Abcam) was infused intracranially 5-min prior to cocaine injection (15 mg/kg i.p.) and behavioral testing.

### Cocaine self-administration

Rats were implanted with subcutaneous pumps delivering haloperidol (0.5 mg/kg/d, Alzet) for 14 days. On day 14, pumps were removed and animals were fitted with intrajugular catheters. Seven days after recovery from surgery, haloperidol pretreated rats and untreated controls began daily 2 h cocaine self-administration sessions, where active lever presses were paired with light and tone cues for 5 s and cocaine delivery (0.4 mg/kg/infusion). Inactive lever presses had no consequence. After 10 days of self-administration, rats underwent extinction training (2 h/d), where cues and cocaine delivery were withheld during active lever pressing. Extinction training continued for 12 days. The following day, rats were returned to the operant chamber and light/tone cue pairings were restored to the active lever during a 60-min session, but no cocaine was delivered.

### Other behavioral testing

Methodological details are provided in the Supplementary materials and methods.

### Statistics

Transformation of Ca^2+^ events into binary values was conducted using MATLAB and cluster analysis and relative data representations were performed using SAS statistics. Data were analyzed using a Student’s *t* test or one- or two-way ANOVA with repeated measures when possible. A Pearson’s correlation coefficient was calculated to determine the relationship between Ca^2+^ events and locomotion. In all cases, statistical significance was set at *p* < 0.05.

## Results

### Neurobiology of antipsychotic-induced behavioral supersensitivity

#### Induction of behavioral supersensitivity

The symptoms of antipsychotic-induced behavioral supersensitivity can occur during ongoing antipsychotic treatment [22–24], but are exacerbated by treatment discontinuation [7–10], and are best modeled in rodents during locomotor sensitization to psychostimulants after chronic antipsychotic discontinuation [11, 22, 32]. For these reasons, we first tested whether discontinuation from chronic treatment with clinically equivalent doses of haloperidol heightened the psychomotor response to a single cocaine injection in mice. In keeping with clinically valid scenarios, animals underwent continuous haloperidol treatment via osmotic infusion (0.5 mg/kg/d) through the expression of treatment failure (i.e., acquired resistance, 14 days in rodents) [23, 24, 33], a time at which most patients decide to discontinue antipsychotic treatment [6]. Animals then received a single dose of cocaine (15 mg/kg, i.p.) 7 days after haloperidol discontinuation.

**Fig. 1:**
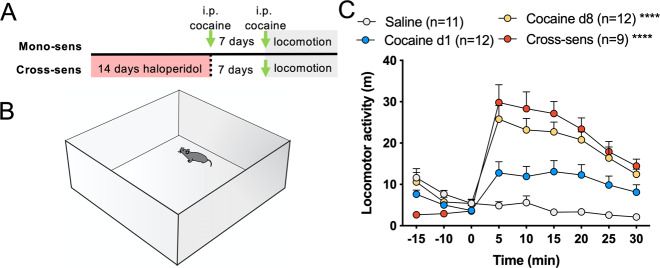
Cocaine-induced locomotor mono- and cross-sensitization. **A**, **B** Schematic of protocols used to induce locomotor sensitization and testing environment. Two cocaine injections (15 mg/kg, i.p.) separated by 7 days abstinence were used to induced locomotor sensitization (Mono-sens). A single cocaine injection (15 mg/kg, i.p.) after 7d discontinuation from chronic haloperidol (i.e., 14 days pretreatment) was used to induce locomotor sensitization (Cross-sens). **C** Mono-sensitized and cross-sensitized mice exhibited enhanced locomotion relative to saline-treated mice (two-way ANOVA Time × Treatment *F*(24,320) = 11.68, *p* < 0.0001, ****p < 0.0001 vs Saline using Tukey’s test). Animal *N* shown in legend.

In separate animals, we induced cocaine sensitization using two repeated cocaine injections (15 mg/kg, i.p.) to compare the sensitized responses to cocaine in the two groups—those pre-exposed to the antipsychotic (cross-sensitization) and those that received only cocaine (mono-sensitization) (Fig. 1A, B). Acute cocaine in animals pretreated with haloperidol enhanced locomotion (Fig. 1C) in a similar manner as in haloperidol-naive animals that received repeated cocaine injections [30], demonstrating behavioral supersensitivity at this timepoint.

#### Behavioral supersensitivity coincided with the loss of D2r-mediated IPSCs

**Fig. 2:**
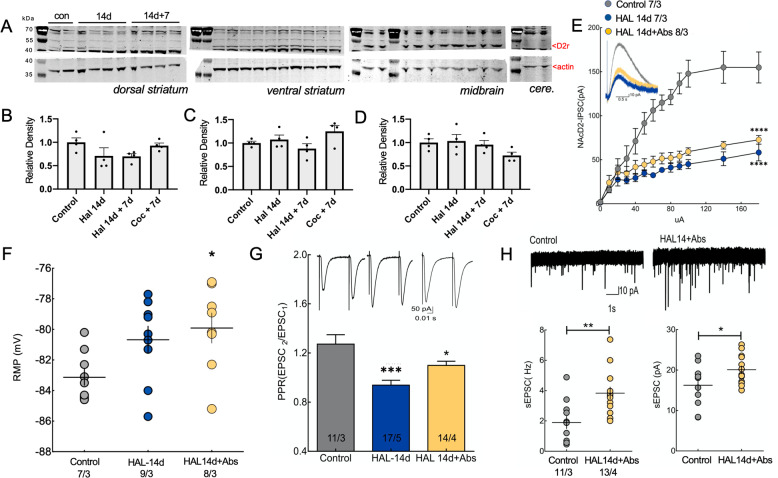
Chronic haloperidol treatment and discontinuation decreased D2r function and enhanced excitatory transmission onto D2-MSNs. **A** Dorsal striatum, ventral striatum, and midbrain tissue extracts were analyzed for D2 receptor expression by western blotting. For each blot, extracts from two control mice are shown, followed by four mice undergoing continuous treatment with haloperidol for 14 days and 4 mice that discontinued haloperidol treatment. The D2r (~50 kDa) was identified based on its absence in tissue extracts from the cerebellum. The same blots were cut and probed for actin (~37 kDa). D2r upregulation was not observed after haloperidol treatment or discontinuation, or 7d after an acute cocaine injection in untreated mice, in tissue extracts from the dorsal striatum (**B** one-way ANOVA *F*(3,12) = 2.078 *p* = 0.1567), ventral striatum (**C** one-way ANOVA F(3,12)=2.572 *p* = 0.1028), or midbrain (**D** 1-way ANOVA *F*(3,12) = 2.017 *p* = 0.1653). **E** D2r-mediated IPSCs were impaired in mice treated with haloperidol for 14d and in mice that discontinued chronic haloperidol treatment (two-way ANOVA Treatment × Current *F*(24,252) = 11.01, *p* < 0.0001, ****p < 0.0001 vs. Control using Dunnett’s test). **F** D2-MSNs were more depolarized at rest after haloperidol discontinuation compared to controls (*t*(13.62) = 2.209, *p < 0.05 using Welch’s test). **G** Chronic haloperidol treatment and treatment discontinuation reduced PPR-evoked EPSCs in D2-MSNs (one-way ANOVA *F*(5,71) = 7.173, *p* < 0.0001, *p < 0.05, ***p < 0.001 vs. Control using Dunnett’s test). (**H**, top panel) Representative traces from D2-MSNs in a control and after haloperidol discontinuation. **H** Frequency (left panel, *t*(22) = 3.222, **p = 0.0039) and amplitude (right panel, *t*(22) = 2.140, *p = 0.0437) of spontaneous excitatory postsynaptic currents were increased after haloperidol discontinuation. In **B–D** scatter shows animal *N*. In **E–H** N shown as cells/animals in legend or bars. Haloperidol 14 days, HAL 14d; Haloperidol discontinuation, HAL 14d + Abs.

The most commonly cited cause of antipsychotic-induced behavioral supersensitivity is upregulated D2r expression after discontinuation of long-term antipsychotic treatment [7]. To test whether the D2r was upregulated in our model of behavioral supersensitivity, we examined D2r levels in the ventral striatum, dorsal striatum, midbrain, and cerebellum by western blot after 14 days of continuous haloperidol treatment, after 7 days of treatment discontinuation, and in control conditions. We found no change in D2r expression in either antipsychotic-treated group, or in animals that received a single cocaine injection followed by 7 days abstinence, relative to untreated control animals (Fig. 2A–D).

D2r is a metabotropic receptor coupled to G_i/o_ intracellularly that, when stimulated by dopamine, reduces cAMP production and cell excitability via the opening of the rectifier potassium channel GIRK2. In absence of D2r expression changes, we tested for a compensatory increase in D2r sensitivity after antipsychotic treatment. To do this, we overexpressed GIRK2 in the NAcore of D2-cre mice and measured dopamine D2r-dependent IPSCs as a sensor of D2r activation [26, 34, 35]. Interestingly electrical stimulation of dopamine terminals did not evoke D2r-dependent IPSCs during antipsychotic treatment or after treatment discontinuation (Fig. 2E), indicating a loss of function of the D2r to trigger its constitutive intracellular G_i_ inhibitory signaling. These data suggest that neither D2r expression nor putative increased function of the receptor can account for the expression of antipsychotic-induced behavioral supersensitivity.

#### Enhanced excitatory transmission on D2-MSNs during antipsychotic discontinuation

MSN excitability relies heavily on potassium-dependent intrinsic mechanisms and convergent glutamatergic inputs [36], which are both regulated by the D2r [36, 37]. Given the reduced D2r function in antipsychotic pretreated animals (Fig. 2E), we hypothesized that D2-MSNs would display a more depolarized resting membrane potential (RMP) and an augmented glutamate transmission after antipsychotic treatment. Accordingly, the RMP of D2-MSNs was depolarized in animals discontinuing the antipsychotic compared with controls (Fig. 2F).

Excitatory neurotransmission is altered by antipsychotic drugs at therapeutic equivalent doses, in part through increased recycling and readily releasable synaptic vesicle pools [24, 38]. Importantly, the machinery involved in synaptic vesicle trafficking is fundamental for the expression of short-term plasticity, such as neurotransmitter release facilitation [39]. To assess changes in glutamate transmission and relative neuroplasticity, we first examined whether glutamate release probability was altered by haloperidol discontinuation by measuring spontaneous excitatory postsynaptic currents (sEPSCs) and the paired-pulse ratio (PPR) of evoked excitatory postsynaptic currents (eEPSCs) in the NAcore. PPR was reduced on D2-MSNs during ongoing haloperidol treatment and after its discontinuation (Fig. 2G) and both frequency and amplitude of sEPSCs were increased after discontinuation (Fig. 2H), confirming both pre and postsynaptic potentiation of excitatory transmission onto NAcore D2-MSNs. No such changes were observed in NAcore D1-MSNs after haloperidol discontinuation (Fig. S1).

**Fig. 3:**
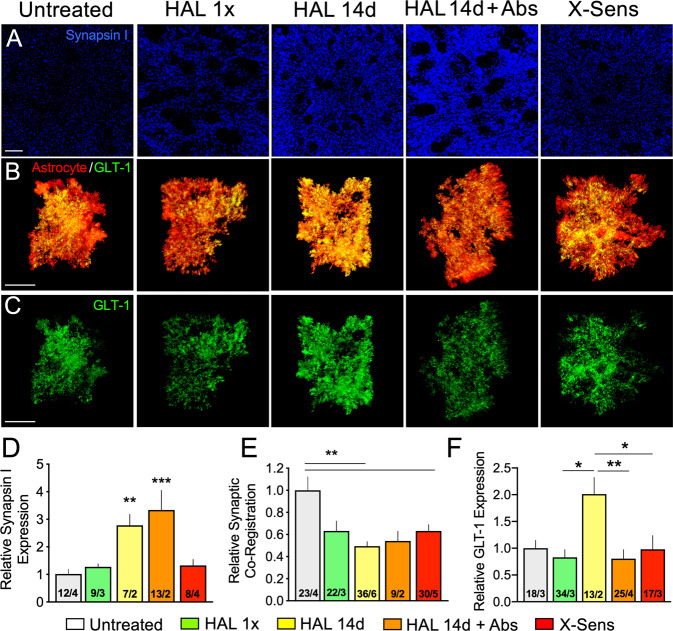
Haloperidol discontinuation produced presynaptic and astroglial changes that facilitate excitatory transmission. **A**–**C** Confocal microscopy was used to image immunoreactive Synapsin I (blue) and GLT-1 (green) in association with virally-labeled astroglia (red) in the NAcore of mice after haloperidol treatment. **D** Synapsin I immunoreactivity was quantified as a measure of non-dopaminergic resting pool vesicles at terminals in the NAcore and was found to be significantly elevated after chronic haloperidol treatment with or without discontinuation (Abs) (Kruskal–Wallis = 36.05, *p* < 0.0001). **E** Synaptic proximity of NAcore astroglia, measured as co-registration of the astroglial membrane with Synapsin I, was reduced during long-term haloperidol treatment (Kruskal–Wallis = 16.11, *p* = 0.0065). **F** GLT-1 expression was elevated during chronic haloperidol treatment, but was significantly downregulated after 7d of discontinuation (Abs), despite elevated levels of Synapsin I (Kruskal–Wallis = 13.85, *p* = 0.0078). Scale = 15 µm in (**A**–**C**). *N* shown in bars as stacks/animals in (**D**) and cells/animals in (**E**, **F**). *p < 0.05, **p < 0.01, ***p < 0.001 using Dunn’s post hoc test. Acute haloperidol, HAL 1×; Haloperidol 14 days, HAL 14d; Haloperidol discontinuation, HAL 14d + Abs; Cross-sensitization, X-Sens.

The initial release probability (i.e., first pulse of a train) of presynaptic excitatory transmission is directly related to vesicle pool size [39–41], and is regulated by Synapsin I, a prominent protein kinase substrate [41, 42] associated with readily releasable vesicles and with recycled synaptic vesicles following synaptic activity [43]. To validate our ex vivo finding, we used confocal microscopy and found increased density of Synapsin I-positive puncta in the NAcore after haloperidol discontinuation (Fig. 3A, D), indicating presynaptic changes consistent with increased vesicle pool size and supporting elevated glutamate release capacity at the first pulse (Fig. 2H). The injection of a single dose of cocaine reduced Synapsin I levels within 15 min, supporting a relationship between increased Synapsin I expression and increased transmitter release capacity during behavioral supersensitivity.

The extracellular concentration of glutamate is regulated perisynaptically by astrocytes via their synaptic proximity and expression of the glutamate transporter GLT-1 that regulates glutamate uptake following synaptic release [44]. Both synaptic proximity of astroglial processes and surface diffusion of GLT-1 are dynamic and impact autoinhibitory control of excitatory transmission and postsynaptic excitation [45–49]. We measured changes in synaptic proximity of NAcore astroglia by quantifying confocal co-registration of mCherry-labeled astrocytes and immunoreactive Synapsin I. Synaptic co-registration of the astroglial membrane was reduced after chronic haloperidol treatment and discontinuation (Fig. 3E) consistent with synaptic retraction of astrocyte processes during behavioral supersensitivity. Synaptic insulation by astroglial processes permits efficient glutamate uptake through perisynaptic proximity of GLT-1 [44]. Moreover, basal glutamate is regulated by astroglia that maintain tone on presynaptic autoreceptors via cystine-glutamate exchange [47]. Thus, retraction of astroglial processes is expected to disinhibit glutamate release, permit glutamate spillover and facilitate synaptic recruitment [46, 50]. We also found that GLT-1 expression increased during haloperidol treatment, but returned to baseline after treatment discontinuation, despite persistent increases in Synapsin I (Fig. 3F). The reduced synaptic proximity of astroglial processes that harbor GLT-1 paired with its relative downregulation after haloperidol discontinuation would be expected to increase and/or prolong excitatory transmission during behavioral supersensitivity.

**Fig. 4:**
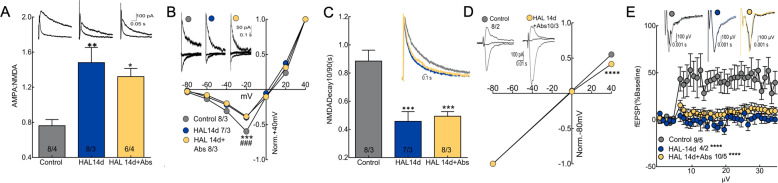
Chronic haloperidol treatment and discontinuation induced excitatory plasticity on D2-MSNs. **A** Isolation of AMPAr and NMDAr currents in D2-MSNs revealed increased AMPAr:NMDAr during chronic haloperidol treatment and after treatment discontinuation (one-way ANOVA *F*(2,21) = 7.944, *p* = 0.0027, *p < 0.05, **p < 0.01 vs. Control using Dunnett’s test). **B** NMDA I/V curves were reduced in D2-MSNs during haloperidol treatment and after treatment discontinuation (two-way ANOVA Treatment × Voltage *F*(12,147), *p* < 0.0001, ***p < 0.001 vs. HAL 14d, ^###^*p* < 0.001 vs HAL 14d + Abs using Dunnett’s test). **C** NMDA currents in D2-MSNs decayed faster during chronic haloperidol treatment and following treatment discontinuation (one-way ANOVA *F*(2,20) = 14.59, *p* = 0.0001, ***p < 0.001 vs. Control using Dunnett’s test). **D** Rectification index I/V plots of normalized AMPAr eEPSCs from control mice and mice that discontinued haloperidol treatment. Inset shows representative traces of eEPSCs recorded from −80, 0, and +40 mV. Animals that discontinued haloperidol treatment displayed stronger rectification than controls (two-way ANOVA Treatment *F*(1,48) = 8.460 *p* < 0.0055 vs. Control, ****p < 0.0001 using Bonferroni’s test). **E** High-frequency stimulation increased field EPSPs from control animals, but not from animals undergoing chronic haloperidol treatment or animals that discontinued treatment (two-way ANOVA Treatment *F*(2,680) = 149.8, *p* < 0.0001, ****p < 0.0001 vs. Control using Tukey’s test). In **A**–**D**, *N* shown in bars or legends as cells/animals. Haloperidol 14 days, HAL 14d; Haloperidol discontinuation, HAL 14d + Abs.

Increased glutamatergic transmission leads to postsynaptic plasticity, commonly measured as an increase in current across α -amino-3-hydroxy-5-methyl-4-isoxazolepropionic acid receptors (AMPArs) on dendritic spines, and relatively stable expression of N-methyl-D-aspartate receptors (NMDArs), resulting in changes in the ratio of synaptic currents through AMPArs and NMDArs [51–53]. Pharmacological isolation of AMPAr and NMDAr currents revealed an increased AMPA/NMDA receptor ratio in D2-MSNs (Fig. 4A), but not D1-MSNs during haloperidol discontinuation (Fig. S1B). This change was due to both a decrease in NMDAr-dependent currents in D2-MSNs (Fig. 4B, C) and an increase in currents through Ca^2+^-permeable AMPArs (Fig. 4D), receptors that regulate several forms of synaptic plasticity [54].

We next evaluated the AMPAr component of evoked EPSCs in NAcore D2-MSNs from mice pre-exposed to haloperidol and found that EPSCs at negative potentials were larger than those measured at positive potentials after antipsychotic treatment (Fig. 4D, insets). The *I*–*V* relationship shown in Fig. 4D confirms the rectification of EPSCs after pre-exposure to antipsychotic, but not in control conditions, and confirms insertion of Ca^2+^-permeable AMPArs during behavioral supersensitivity. Of note, since we obtained rectification of AMPAr currents using the weak channel blocker spermine (100 μM), we did not find it necessary to replicate the same result using a more potent channel blocker such as NASPAM [55]. We then applied high-frequency stimulation (HFS) of glutamatergic afferent fibers in the NAcore to trigger long-term potentiation (LTP) and found that HFS induced LTP in control tissue as expected, but not in tissue from antipsychotic-treated mice (Fig. 4E), suggesting occlusion of LTP.

#### D2-MSN hyperactivity during behavioral supersensitivity

**Fig. 5:**
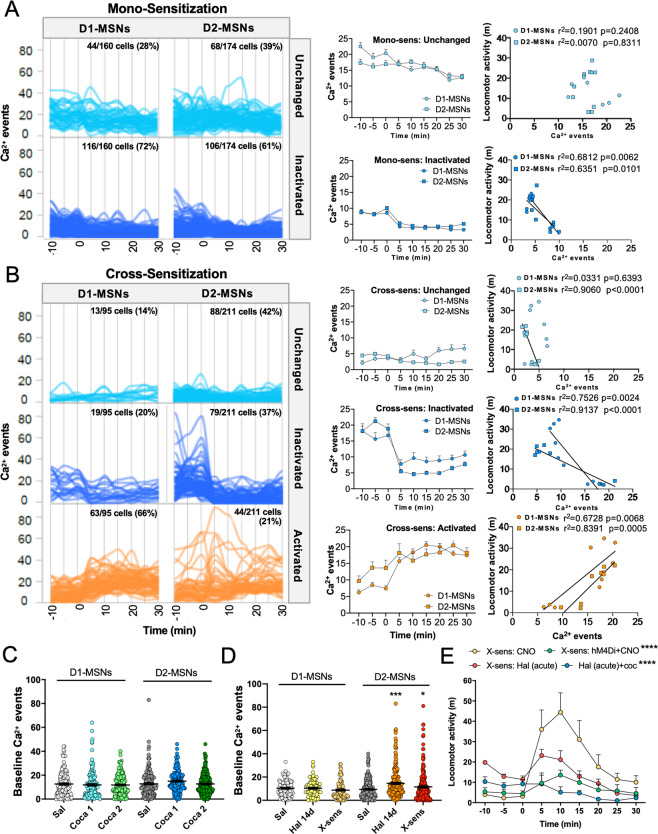
Hyperactive D2-MSNs drive behavioral supersensitivity. Unsupervised k-means clustering was used to identify clusters of cells that were unchanged, inactivated, or activated by acute cocaine delivery (time 0) in animals undergoing mono- (**A**) or cross-sensitization (**B**). Ca^2+^ events in D1- or D2-MSNs over time shown as individual cells (left panel) or group means (middle panel; Mono-sens: *Unchanged*, two-way ANOVA Time *F*(4.477,492.5) = 12.98 *p* < 0.0001, *p* = 0.2712 0 vs. 5 min using Dunnet’s test; *Inactivated*, two-way ANOVA Time *F*(3.430, 754.6) = 55.74 *p* < 0.0001; Cross-sens: *Unchanged*, two-way ANOVA Time *F*(4.987,493.8) = 2.056 *p* = 0.0698; *Inactivated*, two-way ANOVA Time *F*(3.216, 308.7) = 35.15 *p* < 0.0001; *Activated*, two-way ANOVA Time *F*(4.074, 427.7) = 34.65 *p* < 0.0001). Right panel shows relationship between D1- and D2-MSN activity and locomotion averaged across animals in each time bin. **C** No difference was observed in D1- or D2-MSN activity during the baseline recording period before saline injection or the first or second cocaine injection in mono-sensitized mice (two-way ANOVA Treatment *F*(2,953) = 1.279 *p* = 0.2789). **D** Baseline D1-MSN activity was unchanged by continuous haloperidol treatment (Hal 14d) or haloperidol discontinuation (X-sens). Instead, D2-MSN activity was elevated during continuous haloperidol treatment and during treatment discontinuation (2-way ANOVA Treatment *F*(2,475) = 4.088 *p* = 0.0174, *p < 0.05, ***p < 0.001 vs. Sal using Fisher’s test). **E** Stimulation of an inhibitory G_i_-DREADD in NAcore D2-MSNs prevented locomotor cross-sensitization (green) compared to animals not expressing the DREADD (yellow, two-way ANOVA Time × Treatment *F*(24,88) = 7.220 *p* < 0.0001, ****p < 0.0001 vs. Cross-sens: CNO). Notably, during cross-sensitization, acute haloperidol (red) was insufficient to blunt cocaine-induced hyperlocomotion compared with untreated animals (blue), ****p < 0.0001 vs. Cross-sens: Hal (acute). In **A**–**E**, *N* = 5 mice/grp (D2-MSNs) or 3–5 mice/grp (D1-MSNs).

Based on our findings of enhanced D2-MSN excitability ex vivo, we next sought to determine whether NAcore D2-MSN activity could be linked to behavioral supersensitivity in vivo. Spontaneous and sensitized locomotion and goal-directed behaviors are orchestrated by activity of D1- and D2-MSNs in the NAcore [27–29, 56, 57]. To examine the cellular basis of locomotion during the expression of antipsychotic-induced behavioral supersensitivity, we recorded single-cell Ca^2+^ dynamics in 1852 MSNs in the NAcore in vivo (Fig. S2, Table S1) during locomotor responses to cocaine in cross-sensitized (892 cells, Fig. S3B) and mono-sensitized (960 cells, Fig. S3A) mice. Unsupervised k-means clustering revealed the underlying response structure of D1- and D2-MSNs in sensitized mice (Fig. S3C–F). Clusters with similar patterns over the recording session were combined and classified as *unchanged*, showing stable activity before and after i.p. cocaine, *inactivated*, showing depression of Ca^2+^ events in response to cocaine, or *activated*, showing increased Ca^2+^ activity after cocaine injection (Fig. 5A, B).

While MSNs were *unchanged* or *inactivated* in mono-sensitized mice (Fig. 5A), we found a subgroup of both D1- (66%) and D2- (21%) MSNs that were *activated* by a single dose of cocaine in cross-sensitized mice (Fig. 5B). Notably, the baseline activity of D2-MSNs was selectively enhanced relative to control conditions during ongoing chronic haloperidol treatment and after its discontinuation (Fig. 5D), consistent with our detection of enhanced excitatory plasticity in these cells. This increase in baseline activity was not observed in mono-sensitized animals and no change in baseline activity of D1-MSNs was observed in either mono- or cross-sensitized mice (Fig. 5C, D). When cell clusters were examined separately regarding their relationship to locomotor output, we found that unchanged and decreased MSNs in mono-sensitized and cross-sensitized mice either did not correlate or correlated negatively with locomotion (Fig. 5A, B, right panel). Instead, both D1- and D2-MSNs that were activated by cocaine in cross-sensitized mice correlated positively with locomotion (Fig. 5B, right panel), suggesting a role for hyperactive MSN subpopulations in mediating behavioral supersensitivity. Interestingly, baseline activity of MSN subclusters after haloperidol discontinuation served as a reliable predictor of cellular responses to cocaine during cross-sensitization, in that higher baseline activity predicted depression of cellular response to cocaine and lower basal D2-MSN activity predicted cocaine-induced hyperactivity (Fig. S4).

#### Restoration of G_i_ intracellular signaling reversed behavioral supersensitivity

To assess the functional role of D2-MSN hyperactivation in the expression of behavioral supersensitivity, we virally delivered a G_i_-coupled designer receptor activated by the designer drug (DREADD) CNO [58] to D2-MSNs in the NAcore of haloperidol pretreated mice (Fig. S5). G_i_-DREADD expression would be expected to restore G_i_-dependent IPSCs in D2-MSNs after haloperidol discontinuation. Mice that received CNO intracranially to inhibit D2-MSN activity did not exhibit locomotor cross-sensitization after cocaine injection compared with mice not expressing the G_i_-DREADD (Fig. 5E). Thus, silencing hyperactive D2-MSNs suppressed locomotor cross-sensitization during behavioral supersensitivity. Locomotion in mice undergoing DREADD inhibition in D2-MSNs was comparable to locomotion in untreated mice that received acute haloperidol to suppress cocaine-induced locomotion (Fig. 5E), further confirming that suppression of NAcore D2-MSN activity is necessary for antipsychotic efficacy as observed in acute administration paradigms [26].

### Behavioral symptoms of antipsychotic-induced behavioral supersensitivity

#### Antipsychotic-treatment resistance after discontinuation

Discontinuation of chronic antipsychotic treatment is a risk factor for diminished antipsychotic efficacy when treatment is reinstated (i.e., antipsychotic treatment resistance) [7–10]. We assessed the efficacy of an acute injection of haloperidol (0.5 mg/kg, i.p.) to inhibit hyperlocomotion induced by a single cocaine injection (15 mg/kg, i.p.) in mice naive to the antipsychotic and in mice that discontinued antipsychotic treatment (as in Fig. 1C). We found that while haloperidol effectively blocked the locomotor response to cocaine in animals naive to antipsychotic treatment, its efficacy was very much decreased in mice that had undergone antipsychotic discontinuation (Fig. 5E), consistent with treatment resistance after antipsychotic discontinuation.

#### VCMs were not observed in mice or rats after antipsychotic discontinuation

The occurrence of neurological symptoms such as dyskinesias are commonly thought to be associated with antipsychotic-induced behavioral supersensitivity [7, 11]. We assessed if discontinuation from regimens of haloperidol treatment leading to behavioral supersensitivity also caused VCMs, a proxy for oral dyskinesias in mice and rats [59]. While mice displayed no visible dyskinesia at any point, symptoms were present in rats during ongoing treatment, but not after treatment discontinuation (Fig. S6). To compare our own observations with different treatment protocols, we performed a systematic review of the literature on spontaneous VCMs in rodents. We searched PubMed, EMBASE, and Web of Science for relevant studies using search terms for animal studies, antipsychotics, and withdrawal (Fig. S7). We found that spontaneous VCMs are not increased after discontinuation of second-generation antipsychotic drugs, except for high doses of risperidone, whereas high levels of spontaneous VCMs are detected in young and old rats 21 days after discontinuation of prolonged daily haloperidol treatment (75 days) (Fig. S8). Altogether, these data suggest that oral dyskinesia in rodents is more commonly observed after prolonged or high-dose antipsychotic treatment in rodents and may not reliably align with expression of behavioral supersensitivity.

#### Drug-seeking as an emergent consequence of antipsychotic discontinuation

The likelihood of substance use disorder in patients with schizophrenia is ~4.6-fold higher than in the general population [60, 61], especially among treatment non-adherent patients [62]. Interestingly, a small sample of non-psychotic drug-addicted patients was reported to co-abuse antipsychotic medications to enhance the effects of addictive substances [63]. This raises the question of whether discontinuing antipsychotic treatment enhances vulnerability to substance use disorder. Traditional animal models of drug addiction vulnerability involve locomotor sensitization induced by intermittent psychostimulant injections [64–67]. Because haloperidol and cocaine produced locomotor cross-sensitization, we sought to determine whether this response was predictive of additional behavioral features of substance use disorder using the self-administration, extinction, and reinstatement model of cocaine use and relapse.

**Fig. 6:**
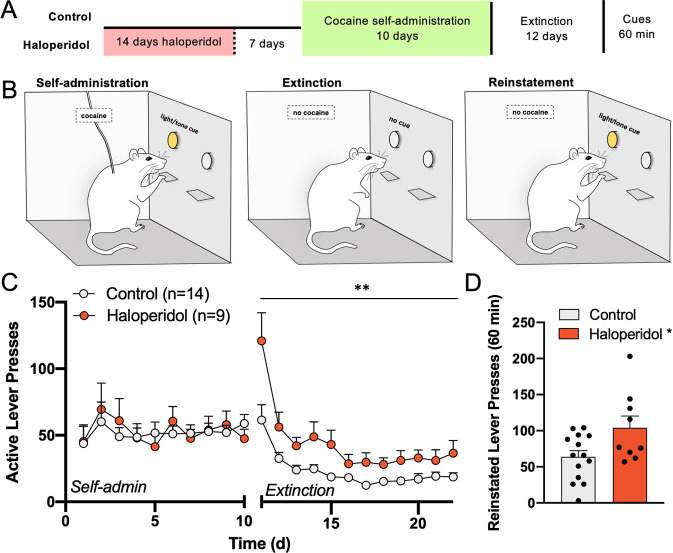
Haloperidol discontinuation exacerbated behavioral features of substance use disorder. **A**, **B** Schematic of treatment protocol and experimental timeline. Untreated rats or rats discontinuing haloperidol (0.5 mg/kg/d s.c. via osmotic infusion) self-administered cocaine for 10 days (2 h/day) and active lever presses for cocaine were paired with light and tone cues (Self-Administration). Next, cocaine and cues were withheld during active lever pressing and lever pressing was extinguished over 12 days (Extinction). The following day cocaine seeking was reinstated using cocaine-associated cues for 60 min (Reinstatement). **C** Haloperidol pretreated rats did not differ from controls in lever pressing during self-administration, but pressed significantly higher on the active lever for the duration of extinction training (two-way ANOVA, Treatment × Time *F*(21,440) = 1.937, **p < 0.008). **D** During reinstatement, haloperidol pretreated rats also pressed higher for cocaine-associated cues in the absence of cocaine delivery (*t*(21) = 2.396, *p = 0.013). Animal *N* shown in (**C**).

We trained male and female rats to self-administer cocaine after abstinence from haloperidol according to the same timeline applied during locomotor cross-sensitization (Fig. 6A). Operant training was conducted on an FR1 schedule, and cocaine delivery was paired with light and tone cues for 2 h each day (Fig. 6B, Self-administration). There were no differences in active or inactive lever pressing between haloperidol pretreated rats and control animals during 10 days of self-administration (Figs. 6C, S9A), suggesting that the acquisition of learned operant responding for cocaine and total cocaine intake were not altered by haloperidol discontinuation. Next, active lever pressing was extinguished by removal of cocaine delivery and cues (Fig. 6B, Extinction). In control animals, active lever presses gradually decreased over time in the absence of drug reward and a stable baseline of responding was observed within 3–5 days of extinction training. In haloperidol-pretreated rats, however, active lever pressing was elevated compared to controls throughout extinction training and baseline responding in the drug-paired context remained elevated despite the absence of the reward (Fig. S9B, Fig. 6C). After 12 days of extinction, animals were returned to the operant box and light/tone pairings were restored to the active lever, but no cocaine was delivered (Fig. 6B, Reinstatement). Cocaine-associated cues stimulated lever pressing in haloperidol-pretreated rats compared to control animals during a 60-min reinstatement test (Figs. 6D, S9C, D), a widely accepted model of cue reactivity [68] linked to drug relapse in human patients [69]. Thus the main deficit emerging in supersensitive animals was surprisingly not an increase in cocaine intake as reported in studies applying mono-sensitization procedures [64], but involved disrupted extinction of operant responding (i.e. lever pressing) for cocaine despite its absence. Similarly, haloperidol pretreated animals displayed enhanced measures of seeking in response to drug-associated cues, perhaps owing to a deficit in within-session extinction of behavioral responding in the absence of cocaine (Fig. S9C). Together, these data indicate that antipsychotic discontinuation may produce features of substance use disorder coincident with behavioral supersensitivity and point to antipsychotic discontinuation as a potential underlying factor contributing to the epidemiological observation that substance use disorder is often comorbid with schizophrenia and other psychiatric disorders where patients are medicated with antipsychotic drugs.

## Discussion

### Summary of results

In the present study, we describe the neurobiology underlying antipsychotic-induced behavioral supersensitivity and provide new support for the active role of D2-MSNs in generating motor outputs in response to psychostimulants. Hyperexcitation of NAcore D2-MSNs was the main neuropathology emerging during chronic antipsychotic treatment (14 days, 0.5 mg/kg/d), treatment discontinuation (14 days treatment + 7 days withdrawal), and the expression of behavioral supersensitivity (14 days treatment + 7 days withdrawal + a single cocaine injection, 15 mg/kg i.p.). In ex vivo studies we determined that chronic treatment and discontinuation of clinically relevant doses of haloperidol were characterized by increased glutamatergic transmission onto D2-MSNs, but not D1-MSNs, accompanied by retraction of perisynaptic astrocytic processes and an increase in abundance of non-dopaminergic vesicles presynaptically without a corresponding increase in GLT-1 expression. We also found significant neuroplasticity in D2-MSNs, characterized by insertion of Ca^2+^-permeable AMPArs, increased AMPAr/NMDAr ratio, and occluded LTP. We confirmed D2-MSN hyperexcitation using in vivo Ca^2+^ imaging during and after haloperidol treatment, and during antipsychotic-induced behavioral supersensitivity triggered by acute cocaine. D1-MSNs appeared to have preserved responses to cocaine, although their firing was not enhanced by haloperidol during or after treatment.

In separate animals, we found no increases in D2r density within regions of interest during or after haloperidol treatment and instead we found decreased ability of the D2r to elicit G_i_-dependent IPSCs. When D2r function was restored via activation of G_i_-DREADDs in freely moving animals, the expression of antipsychotic-induced behavioral supersensitivity was abolished. Animals with behavioral supersensitivity exhibited antipsychotic treatment resistance, since reinstated haloperidol treatment did not abolish the supersensitive response to cocaine in animals that had undergone chronic haloperidol pretreatment. We also observed tardive dyskinesia during, but not after chronic antipsychotic treatment. Most importantly, we found that antipsychotic discontinuation is a risk factor for substance use disorder since it impaired extinction of cocaine seeking, and enhanced cue-induced cocaine seeking during withdrawal in a rat model of addiction and relapse.

### Neurobiology of behavioral supersensitivity

Very little is understood regarding the mechanisms by which chronic antipsychotic treatment or its discontinuation cause behavioral supersensitivity. The most common interpretation attributes this side effect to a potentiated D2r response to dopamine following receptor upregulation, and/or to greater expression of the high-affinity D2r isoform [14, 15], both of which have proven to be inconsistent in preclinical and clinical studies. While D2r density can be upregulated with high doses of D2 antagonists in animals according to some studies [20–22], antipsychotic-induced behavioral supersensitivity is more consistently driven by the duration of treatment [22–24] and can occur without increases in receptor expression [16], including during ongoing treatment [22–24] while D2rs are substantially blocked [24].

We extend these previous observations by showing no upregulation of the D2r in the ventral striatum, dorsal striatum, or midbrain during chronic haloperidol treatment or after its discontinuation. Interestingly, there was a trend toward decreased expression of the receptor in the ventral striatum during behavioral supersensitivity. To measure potential changes in the sensitivity of the D2r to dopamine, we overexpressed the GIRK2 channel, which opens to hyperpolarize the neuronal membrane when dopamine stimulates the D2r [26, 34, 35]. Against our expectations, dopamine release, evoked by electrical stimulation of dopamine terminals, was unable to generate D2r-dependent G_i_-mediated IPSCs in D2-MSNs overexpressing GIRK2. These data suggest that behavioral supersensitivity induced by antipsychotics is not related to increased D2r expression or function. Instead, when D2r function was effectively restored in D2-MSNs by activation of G_i_-DREADDs in freely moving animals, this intervention *prevented* the expression of antipsychotic-induced behavioral supersensitivity.

Taken together, our data indicate that the neurobiological pathway to antipsychotic-induced supersensitivity specifically involves enhanced excitability of D2-MSNs in the ventral striatum. NAcore MSNs are less excitable than cortical neurons and their excitability is strongly regulated by glutamate neurotransmission. Accordingly, we show multiple measures of enhanced excitatory transmission converging selectively onto D2-MSNs in the NAcore and involving pre, post, and perisynaptic adaptations. The imaging studies performed in vivo confirm this profound excitatory plasticity impacting NAcore D2-MSNs as a pathology underlying antipsychotic-induced behavioral supersensitivity. Clusters of D2-MSNs were hyperactivated during chronic haloperidol treatment, after its discontinuation, and also in response to a single cocaine injection. Indeed, after haloperidol discontinuation, cocaine excited 21% of D2-MSNs, a response predicted by lower basal D2-MSN activity. While we do not address the mechanisms involved in triggering this glutamatergic dysregulation, based on our previous work [23, 24, 70, 71], we predict that hyperexcitation of D2-MSNs following antipsychotic treatment likely results from a lack of modulatory dopaminergic input, which is reduced after chronic antipsychotic treatment [23, 24], rendering D2-MSNs hyperexcitable through incoming excitatory transmission. We obtained confirmation of the causality of D2-MSN hyperexcitability in behavioral supersensitivity since chemogenetic restoration of G_i_ signaling in D2-MSNs was sufficient to prevent the supersensitive response to cocaine. Thus, we show that behavioral supersensitivity is characterized by an enduring sensitivity of D2-MSNs to glutamate, exacerbated by an *insensitivity* to dopamine, contrary to the oft-cited D2r-mediated mechanism.

### A role for NAcore D2-MSNs in motivated behavior

These data invite us to rethink the canonical role played by accumbens D2-MSNs in motor outputs. It is commonly thought that stimulation of D1 and D2 receptors with either dopamine or with direct and indirect agonists produce stimulation and inhibition of MSNs via the activation of intracellular G_s/o_ or G_i/o_ proteins, respectively. On the contrary, we not only show that NAcore D1-MSNs were not activated by cocaine during mono-sensitization, but also that silencing D2-MSNs inhibited locomotor activity in mice discontinuing antipsychotic treatment during cross-sensitization, revealing a subpopulation of D2-MSNs that can be triggered to drive hyperlocomotion. Furthermore, it is important to note that when MSN activity during cross-sensitization was averaged, D1- and D2-MSNs were increased and decreased in response to cocaine, respectively, in line with canonical expectations of the impact of cocaine on MSN activity (Fig. S10), though this “canonical” outcome was not observed in the mono-sensitized group. Importantly, while some D2r expression is expected from cholinergic interneurons in the NAcore [72], hyperactive D2r-expressing cells in our study must necessarily be MSNs since they are 21% of labeled cells and cholinergic neurons are ~1% of neurons in the NAcore [73]. Moreover, cholinergic neurons were excluded from electrophysiological recordings based on their morphology [26, 73].

Previous observations by others have explored the contribution of ongoing antipsychotic treatment to drug or food consumption [74, 75], but ours is the first to show that antipsychotic discontinuation leads to increased drug seeking through disrupted extinction of operant responding in drug-associated contexts and after exposure to drug-associated cues. Importantly this model has high face and construct validity for human drug addiction [76]. Indeed, deficits in behavioral extinction and increased reinstated cocaine seeking in rodents would be expected to translate to increased drug relapse rates and shorter abstinence periods in humans undergoing parallel pharmacological treatments. These side effects coincide with long-lasting excitatory plasticity on D2-MSNs, but not D1-MSNs in the NAcore. Since D2-MSNs have been shown to mediate both aversion and reward [77], increased lever pressing during extinction and reinstatement may derive from the enduring motivation of subjects to either reduce aversion during antipsychotic discontinuation or to achieve reward. Accordingly, although not true for all antipsychotics [78], it has been reported that antipsychotic treatment can induce negative symptoms in patients with psychosis [79] and in healthier volunteers [80],

### Relevance for antipsychotic treatment in human patients

The presented data are of fundamental clinical importance since they describe the neurobiology of antipsychotic-induced behavioral supersensitivity and provide a description of the associated behavioral symptoms. Notably, our data show that antipsychotic discontinuation predisposes to antipsychotic resistance when treatment is reinstated and provides a mechanism to interpret previous observations on glutamate alterations in treatment-resistance patients [81]. We also discovered that antipsychotic discontinuation is likely a risk factor for substance use disorder in the absence of schizophrenia. Since we used animals that exhibited no symptoms of psychosis, our data describe neural adaptations induced pharmacologically by antipsychotics and therefore are relevant for patients with and without psychosis. Accordingly, since antipsychotic drugs are widely prescribed off-label [1], we propose the neurobiology and behavioral symptoms described in this study may affect both populations equally, a hypothesis we are currently testing.

While the focus of this report is on the mechanisms underlying antipsychotic-induced behavioral supersensitivity after treatment discontinuation, it should be noted that all of the measures of neuroplasticity observed in animals discontinuing haloperidol were also observed in animals during ongoing treatment. Therefore the mechanisms described in this report may help to interpret not only behavioral supersensitivity occurring after antipsychotic discontinuation, but also during ongoing treatment. These data might also suggest that behavioral supersensitivity induced by antipsychotic treatment is a progressive disorder beginning during treatment and worsening over time, independent from discontinuation.

In conclusion, the presented data reveal specific neurobiological mechanisms driving behavioral supersensitivity, a first step in identifying methods to treat it. While our data warn against adverse effects associated with long-term antipsychotic treatment and discontinuation, they also point to potential strategies to overcome them. For instance, incorporation of deep brain or transcranial magnetic stimulation at low frequencies [82, 83, 84] could be tested as a strategy to reduce or prevent excitatory plasticity on D2-MSNs, which we have shown to be causal in this disorder.

## Supplementary information

Supplemental Material

Supplementary Figure 1

Supplementary Figure 2

Supplementary Figure 3

Supplementary Figure 4

Supplementary Figure 5

Supplementary Figure 6

Supplementary Figure 7

Supplementary Figure 8

Supplementary Figure 9

Supplementary Figure 10

Supplementary Table 1

## Data Availability

All experimental data are available in the main text or within the supplement.
