# Supplementary material for: Accumbens D2-MSN hyperactivity drives antipsychotic-induced behavioral supersensitivity
Source: Mol Psychiatry. 2021 Aug 4;26(11):6159–69. doi: 10.1038/s41380-021-01235-6 (PMC8760070; doi:10.1038/s41380-021-01235-6)
Supplement: Supplementary file 1 — Supplemental Material [file 41380_2021_1235_MOESM1_ESM.docx]

**Supplementary Materials and methods**

**Animals:** We used male and female 8-13-week-old transgenic (tg) mice and Long Evans rats bred in house at the Medical University of South Carolina for all experiments. Original colonies of D1- and D2-Cre BAC and Drd2-eGFP tg lines were generated and gifted by the GENSAT project (Rockefeller University) ^1^. Colonies were maintained by outbreeding tg mice with wild-type C57BL/6J mice (The Jackson Laboratory). Animals were group housed when possible on a reverse 11:13 hour light:dark cycle (8pm-7am) in a humidity- and temperature-controlled environment and had unrestricted access to food and water. Animal body weights ranged from 23-28g (mice) or 150-350g (rats) at the start of experimentation. All procedures involving the use of animals were conducted in accordance with guidelines established by the National Institutes of Health and were approved by the Institutional Animal Care and Use Committee at the Medical University of South Carolina.

**Drugs:** Haloperidol (0.5 mg/kg/d, MilliporeSigma, USA) was dissolved in sterile water containing 0.2 % ascorbic acid and 10% β*-*cyclodextrin (pH ~6) and infused subcutaneously at a constant flow rate of 0.5 µl/h in mice and 5 µl/h in rats over a period of 14 days using Alzet osmotic pumps to obtain striatal D2 receptor occupancy within the therapeutic range for humans (65-80 %) as described in ^2^. In the DREADD study a group of mice received haloperidol intraperitoneally. For i.p. delivery, haloperidol (0.5mg/kg) was dissolved in physiological saline and mildly heated to optimize solubility. Haloperidol was given at a final volume of 10 mL/kg at room temperature in mice. Cocaine (15 mg/kg) was dissolved in sterile physiological saline (0.9%) and administered intraperitoneally at a final volume of 10 mL/kg at room temperature in mice or intravenously at 0.4 mg/kg/infusion in rats during self-administration.

**Virus injections:** Animals were anesthetized with isoflurane (5% for induction and 1-2% for maintenance) and stereotaxic surgeries were conducted using aseptic conditions with a digital stereotaxic instrument (Kopf Instruments, USA). ***In vivo* calcium imaging:** To achieve selective expression of the Ca^2+^-sensing fluorophore GCaMP6f in D1- and D2-MSNs in the nucleus accumbens core (NAcore), D1- and D2-cre mice received injections of AAV1.hSyn.DIO.gCaMP6f (300 nL, Addgene) into the NAcore (AP: +1.6, ML: +1.1, DV: −4.4) at a flow rate of 0.05 μl/min. Following virus injection, a gradient refractive index lens (7.3 mm by 0.6 mm; Inscopix) was implanted above the NAcore (DV: −4.14) as previously described ^3^. GCaMP6f expression was evaluated by visual inspection of fluorescence under light isoflurane anesthesia starting 22 days after virus injection and lens implantation. A baseplate (Inscopix) to host a miniature microscope was secured with black dental cement in those mice showing dynamic GCaMP6f fluorescence generated by Ca^2+^ transients during inspection through the camera. Dust caps were placed on baseplates when mice were in their home cages. **G_i_-DREADD expression in D2-MSNs:** Mice were implanted with bilateral cannulae (Plastics One) situated above the NAcore (+1.6 mm AP, ± 1.1 mm ML, -3.0 mm DV) along with osmotic pumps for subcutaneous infusion of haloperidol. AAV2.FLEX.hM4Di.mCherry (Addgene) or sterile saline (0.5 μL/hemisphere) were delivered 1.4 mm beyond the tip of the guide cannulae at 0.05 μL/min with 5 min diffusion. **Astrocyte membrane label:** Mice received bilateral NAcore injections of AAV5.GFAP.hM3d.mCherry (University of Zurich) using the coordinates and delivery parameters described for GCaMP6f delivery. Virus incubation occurred over the course of 3 weeks. **GIRK2-mediated IPSC recording:** To selectively measure D2r-meditated IPSCs in D2-MSNs by stimulated dopamine transmission, we injected 300 nL of either cre-dependent or -independent AAV2/9.hSynapsin.tdTomato.T2A.mGIRK2-1-A22A.WPRE into the NAcore (AP: +1.6, ML: +1.1, DV: −4.4) in D2-cre or D2-eGFP mice, respectively, as previously described ^4-6^. After viral infusions animals were left in their home cages undisturbed for 3-4 weeks to allow virus expression. In all cases, after experimentation brains were fixed in 4% PFA for histological verification.

**Locomotor sensitization and cross-sensitization.** Male and female 8-13-week-old transgenic mice were group housed (3-5/cage) on a reverse light/dark cycle and had *ad libitum* access to food and water. Mice undergoing cross-sensitization were implanted with subcutaneous osmotic infusion pumps (Alzet) delivering haloperidol at 0.5mg/kg/day for 14 days. Mice were abstinent from haloperidol for an additional 7 days before undergoing locomotor cross-sensitization with cocaine. Pumps were removed 7 days before testing in a subgroup of mice only, since no differences in the behavioral or cellular responses to cocaine in the presence or absence of pumps was observed. Mice undergoing cocaine mono-sensitization received two cocaine injections separated by 7 days incubation as described in ^7^. All mice were habituated in a dimly lit (15 lux) open field apparatus measuring 43 x 43 x 30 cm with mild background noise for 10 min on three consecutive days prior to experimentation. On the last of these 3 days, mice received 0.2 mL physiological saline i.p. to habituate them to physical handling. During experimentation, mice were placed in the open field during 15 min of spontaneous baseline locomotion. Mice were then briefly transferred into the home cage where they received an i.p. injection of cocaine (15mg/kg) before being placed in the locomotor chamber for 30 min of locomotor recording using ANY-maze behavior tracking software (Stoelting).

**Western blotting.** Mice were lightly anesthetized before decapitation and brain extraction. Brains were incubated on ice briefly and dorsal striatum, ventral striatum, midbrain and cerebellum tissue were dissected and manually homogenized in ice-cold RIPA buffer with protease and phosphatase inhibitors (Thermo Fisher) for 20 strokes and then sonicated for 60 s. Samples were centrifuged at 10,000 x g for 10 min. Supernatants were isolated and protein content was quantified using a BCA protein assay (Thermo Fisher). 70 µg of protein was loaded per lane in Criterion Bis-Tris gradient gels (Bio-Rad). Proteins were then transferred onto a PVDF membrane (Bio-Rad) overnight at 4C. Blots were cut horizontally at 40 kDa and blocked in Odyssey blocking buffer (LI-COR) for 1h at room temperature before overnight incubation in primary antibodies (anti-actin, ab95437, Abcam or anti-D2r, AB5084P, EMD Millipore) in block at 4C. After rinsing, blots were incubated in secondary antibodies (LI-COR) at room temperature for 1h and imaged using a LI-COR Odyssey imaging system. The band corresponding to the D2r was identified based on its molecular weight (~50 kDa) and its absence in a blot containing tissue extracts from the cerebellum. Images were converted to grayscale and quantified using FIJI. D2r bands were normalized to actin from the same lane and ultimately to control values from the same blot.

**Confocal microscopy.** 3 weeks before sacrifice, mice received NAcore injections (+1.6 mm AP, +1.1 mm ML, -4.4 mm DV) of a virus used to label the astroglial membrane (AAV5.GFAP.hM3d-mcherry, University of Zurich) under isoflurane anesthesia. Mice were anesthetized using i.p. ketamine and perfused transcardially with 1X phosphate buffer (10 mL) and 4% PFA (20 mL) before brain extraction. Brains were post-fixed for 24h in 4% PFA before slicing at 100 µm using a vibrating blade microtome (Leica). Sections containing the NAcore were permeabilized in 1X PBS with 2% Triton X-100 for 1h at room temperature shaking gently. Tissue was then blocked in 1X PBS with 0.2% Triton X-100 (PBST) and 2% normal goat serum (block) for 1h at room temperature before overnight incubation in primary antibodies (1:1000 anti-GLT-1, ab1783, EMD Millipore and 1:1000 anti-Synapsin I, ab64581, Abcam) in block at 4C. After washing in PBST, tissue was incubated overnight in biotinylated anti-guinea pig antibody (1:1000, Vector Labs) at room temperature in PBST then overnight in fluorescently labeled antibodies (Thermo Fisher) at room temperature in PBST. Tissue was then washed in PBST and mounted onto glass slides. NAcore tissue was imaged using an SP5 confocal microscope (Leica) using the following conditions: 1024 x 1024 frame size, 12-bit resolution, 4-frame averaging, 1-µm step size. Z-stacks were iteratively deconvolved 10 times (Autoquant) and analyzed for fluorescence intensity using Bitplane Imaris. Total Synapsin I expression was quantified in full or partial z-series using the Surface module and normalized to the stack volume. Rendered astroglia were digitally isolated and their co-registration with Synapsin I was normalized to the astroglial volume and to total Synapsin I signal per equivalent frame size to account for changes in astroglial volume and Synapsin I density between groups. GLT-1 expression that co-registered with mCherry was normalized to the astroglial volume. In all cases, data were ultimately normalized to the mean value of the untreated group. Imaging and analyses were conducted blind to animal treatment.

**Acute brain slice preparation:** Mice were deeply anesthetized with isoflurane before rapid decapitation. Brains were quickly removed and placed in ice-cold slicing solution containing (in mM): 92 N-Methyl-D-Glucamine (NMDG); 20 HEPES; 25 glucose; 30 NaHCO_3_; 10 MgCl_2_; 5 ascorbic acid; 3 sodium pyruvate; 2.5 KCl; 1.2 NaH_2_PO_4_; 0.5 CaCl_2_, pH 7.4. Coronal sections (~230 μm) containing the NAcore were obtained using a sapphire blade (Delaware Diamond Knives DDK) in a vibrating blade microtome (Leica, Weltzar, Germany). Slices were then transferred to artificial CSF (aCSF) containing (in mM) 126 NaCl, 2.5 KCl, 26 NaHCO_3_, 2.5 CaCl_2_, 1.5 MgCl_2_, 1.25 NaH_2_PO_4_, and 10 glucose saturated with 95%/5% O_2_/CO_2_ for 30 min at 32°C and were left to incubate at room temperature for at least 30 min before recording. ***Ex vivo* electrophysiology:** Hemisected NAcore slices were submerged in a recording chamber and superfused with 32.5°C aCSF at a flow rate of 2 mL/min. D2- and D1-MSNs from Drd2a-Egfp and Drd2a-Egfp-Drd1a-tdTomato backcrossed reporter mice or Drd2a-cre mice were identified and visualized with a Nikon Eclipse E600FN microscope fitted with infrared differential interference contrast optics. eGFP and TdTomato were visualized by fluorescence using an LED (Thorlabs) through a 40x objective (Nikon, numerical aperture 0.8). Whole-cell electrophysiological recordings were obtained using borosilicate glass microelectrodes (tip resistance, 2.0–4.0 MΩ). In voltage clamp configuration, excitatory post-synaptic currents (EPSCs) were assessed using patch pipettes backfilled with an internal solution containing (in mM): 130 CsMeSO_3_H; 4 NaCl; 2 MgCl_2_; 0.3 EGTA; 10 HEPES; 2 Mg_2_ATP; 0.2 Na_3_GTP; 3 QX314, pH ~7.3 and 280–285 mOsm, whereas D2-mediated inhibitory post-synaptic currents (D2-IPSCs) were measured using pipettes backfilled with (in mM): 115 K-methylsulphate, 20 NaCl, 1.5 MgCl_2_, 10 HEPES(K), 10 BAPTA-tetrapotassium and 1 mg/mL ATP, 0.1 mg/mL GTP, and 1.5 mg/mL phosphocreatine (pH 7.4, 285-290 mOsm). Access resistance was monitored with a 40-ms step of −5 mV and maintained below 20 MΩ, and recordings were accepted for analysis if changes in access resistance were <15%. Electrophysiological signal was amplified using the Multiclamp700A amplifier (Molecular Devices), low-pass filtered at 2 kHz, and digitized at 10 kHz. Data were collected on-line using AxoGraph X (Axo-Graph Scientific) and stored for off-line analysis. All electrophysiology recordings were obtained from NAcore neurons dorsal to the anterior commissure to match Ca^2+^ imaging recordings. **D2 receptor-mediated IPSCs:** Selective recordings of D2r-IPSCs in D2-MSNs were made from GIRK2-expressing D2-MSNs in the NAcore as previously described in ^4-6^ in control conditions, after 14 days haloperidol treatment and after 7d discontinuation from haloperidol. D2-IPSCs were isolated in aCSF that contained 100mM picrotoxin, 300 nM CGP55845, 10mM NBQX, 10mM MK801, 1mM SCH 23390 hydrochloride, 5 mM JNJ16259685, 5mM MTEP, and 200 nM scopolamine hydrobromide to block GABA-A, GABA-B, AMPA, NMDA, D1, mGluR1/5, and M4 mediated synaptic transmission. To evoke D2-IPSCs, MSNs were held at -60mV, and a single stimulation (0.2 ms, 10–160 μA) was used to evoke release from dopamine terminals. **Pre and postsynaptic glutamate transmission:** To evoke post-synaptic currents, afferents were stimulated (100-200 μs) by positioning a bipolar tungsten electrode (FHC Inc.) immediately dorsal (within 150-200 μm) to the cell body. For EPSC paired-pulse ratios, cells were held at -70 mV, and two stimuli were applied at an interval of 50 ms every 20s in the presence of picrotoxin 100μM. The paired-pulse ratio (PPR) was calculated by dividing the amplitude of the second evoked EPSC by the first. To measure the AMPAr:NMDAr ratio cells were held at –70 mV for ~5 min to ensure stability and then were depolarized to +40 mV. Raw EPSC currents then were monitored for 5–10 min at +40 mV, and AMPAr currents were isolated by applying the NMDA receptor antagonist D-2-amino-5-phosphonopentanoate (D-AP5, 50 μM). The NMDA current component was obtained by subtracting the AMPAr current from the total current. The ratio of AMPAr:NMDAr was calculated by dividing the AMPAr over NMDAr currents. Pure NMDAr currents were recorded in the presence of NBQX to block AMPAr currents. To compare the NMDAr I/V relationship using the same activation magnitude, we adjusted the NMDAr peak current in D2-MSNs to ~200 pA at 40 mV. Then in 20mV steps, we changed the membrane potential until reaching -80mV. Each data point on the plot represents the mean of two consecutive observations measured at 0.05 Hz. NMDAr decay kinetics were assessed at +40mV and normalized to the peak current. Spontaneous EPSCs (sEPSCs) were recorded at -75 mV in the presence of picrotoxin 100 μM for 5-10 minutes. The threshold for detection was set to >7 pA, and amplitude and frequency were measured using Axograph and a sliding algorithm template. To measure the AMPAr rectification index, D-AP5 (50μM) was included in the aCSF to block NMDAr currents, and spermine (100μM) was added to the internal solution. AMPAr excitatory postsynaptic currents (eEPSCs) were evoked at -80, 0 and +40 mV at 0.05 Hz, and the rectification index calculated using the equation: RI = EPSC amplitude at -80 mV/EPSC amplitude at +40 mV. Field excitatory post-synaptic potentials (EPSPs) were recorded in the current clamp-mode with an aCSF-filled pipette in the presence of 100 μM picrotoxin. After 5 min of stable baseline, NMDAr-dependent long-term potentiation (LTP) was induced with an established high-frequency stimulation protocol (100 pulses at 100 Hz train of stimuli, 1s duration repeated four times at 20s intervals). To prevent saturation, high-frequency stimulation was delivered at 40-60% of maximal response. Peak field EPSP amplitude was expressed as a percentage of the mean baseline value observed during the 5 min (100%) preceding high-frequency stimulation. Each data point on the plot represents the mean of two consecutive observations measured 30s apart. LTP magnitude was assessed by measuring field EPSP amplitude after induction of high-frequency stimulation. **G_i_-DREADD validation:** To validate that expression of the inhibitory DREADD reduced the excitability of D2-MSNs, current-evoked spike firing (300 pA, 500 ms) was monitored before and during perfusion of the slice chamber with CNO (10 μM).

***In vivo* Ca^2+^ imaging.** Prior to recording, mice were fitted with a head-mounted camera and habituated to the experimental setting for 10-min on three consecutive days as described previously ^6^. During experimentation, Ca^2+^ recordings were temporally synchronized with locomotor activity in the open field by manually launching simultaneous recordings. Grayscale video was recorded using nVista HD Acquisition Software (v.2, Inscopix) at 15 Hz and with 20% LED power. Following data acquisition, images were downsampled 4x and preprocessed for motion correction and regions of interest corresponding to visually identifiable cell bodies were identified using a built-in automated algorithm (Mosaic v. 1.1.2, Inscopix). Ca^2+^ traces from independent neurons were computed based on their spatial and temporal components identified using principal/independent component analysis (PCA/ICA) and confirmed by an investigator blind to animal identifiers. Ca^2+^ events were included if they were ≥ 6x the median absolute deviation of the input trace data and had a minimum mean decay of 0.1 s. Ca^2+^ events were then transformed into binary values, with amplitudes >0 assigned a value of 1 and amplitude ≤0 assigned a value of 0, to obtain the absolute number of spikes per min. Subsequently the sum of spikes per minute for each cell was used to determine spikes in 5 min bins to obtain a total of 9 x 5-min bins. The first 3 bins reflected Ca^2+^ events at baseline (15 min) and the remaining 6 bins represented Ca^2+^ events after saline or cocaine injection (30 min). Next, the absolute number of Ca^2+^ events that passed the sphericity test were categorized to determine distinct cell response patterns using unsupervised K-means clustering for parametric data, which identifies a set of k seeds (i.e. centroids) and assigns each data point to the nearest cluster seed. By reiteration the initial seeds were then replaced by the cluster means and the data points were reassigned. The process continued until no further changes occurred in the clusters. The optimal number of clusters was automatically selected using the fit statistic Cubic Cluster Criterion. Cluster analysis and the relative representations were performed using SAS-JMP statistics. Clusters were categorized empirically as activated, inactivated, or unchanged.

**DREADD inhibition of D2-MSNs.** D2-cre mice were implanted with bilateral cannulae (Plastics One) situated above the NAcore (+1.6 mm AP, ± 1.1 mm ML, -3.0 mm DV) along with osmotic pumps for subcutaneous infusion of chronic haloperidol. Mice then received intracranial injections of AAV2.FLEX.hM4Di.mCherry (Addgene) or sterile saline at 0.5 μL/hemisphere, 0.05 μL/min, with 5 min diffusion 1.4mm beyond the tip of the guide cannulae. Virus incubation occurred during 14 days of haloperidol treatment and 7 days of abstinence following treatment (i.e. 3 weeks). Baseline locomotion was recorded for 15-min before animals were briefly returned to the home cage. Clozapine N-oxide (CNO, 1 mM, 0.3 μL, Abcam) was infused intracranially (0.15 μL/min with 3 min diffusion) with bilateral microinjectors extending 1.4 mm DV beyond the tip of the guide cannulae 5-min prior to cocaine injection (15 mg/kg i.p.) and behavioral testing. Next mice were placed in the open field apparatus for 30-min of locomotor recording. Additional subjects received acute haloperidol (0.5 mg/kg, i.p.) prior to cocaine injection and locomotor recording.

**Quantitation of vacuous chewing movements (VCMs).** Mice or rats were moved into the testing room 1h prior to VCM monitoring and habituated to the testing arena for 5 minutes. VCMs, defined as purposeless mouth opening with or without tongue protrusion, as described in ^8^, were quantified for each animal during 2-min of observation and averaged within treatment groups.

**Meta-analysis.**

To find studies on animal experimentation in PubMed we used the search filter described by Hooijmans et al. (2010) ^9^, the antipsychotics classification described in the WHO ATC classification system ^10^, and terms for withdrawal in the search terms:

(("animal experimentation"[MeSH Terms] OR "models, animal"[MeSH Terms] OR "invertebrates"[MeSH Terms] OR "Animals"[Mesh:noexp] OR "animal population groups"[MeSH Terms] OR "chordata"[MeSH Terms:noexp] OR "chordata, nonvertebrate"[MeSH Terms] OR "vertebrates"[MeSH Terms:noexp] OR "amphibians"[MeSH Terms] OR "birds"[MeSH Terms] OR "fishes"[MeSH Terms] OR "reptiles"[MeSH Terms] OR "mammals"[MeSH Terms:noexp] OR "primates"[MeSH Terms:noexp] OR "artiodactyla"[MeSH Terms] OR "carnivora"[MeSH Terms] OR "cetacea"[MeSH Terms] OR "chiroptera"[MeSH Terms] OR "elephants"[MeSH Terms] OR "hyraxes"[MeSH Terms] OR "insectivora"[MeSH Terms] OR "lagomorpha"[MeSH Terms] OR "marsupialia"[MeSH Terms] OR "monotremata"[MeSH Terms] OR "perissodactyla"[MeSH Terms] OR "rodentia"[MeSH Terms] OR "scandentia"[MeSH Terms] OR "sirenia"[MeSH Terms] OR "xenarthra"[MeSH Terms] OR "haplorhini"[MeSH Terms:noexp] OR "strepsirhini"[MeSH Terms] OR "platyrrhini"[MeSH Terms] OR "tarsii"[MeSH Terms] OR "catarrhini"[MeSH Terms:noexp] OR "cercopithecidae"[MeSH Terms] OR "hylobatidae"[MeSH Terms] OR "hominidae"[MeSH Terms:noexp] OR "gorilla gorilla"[MeSH Terms] OR "pan paniscus"[MeSH Terms] OR "pan troglodytes"[MeSH Terms] OR "pongo pygmaeus"[MeSH Terms])

OR

((animals[tiab] OR animal[tiab] OR mice[Tiab] OR mus[Tiab] OR mouse[Tiab] OR murine[Tiab] OR woodmouse[tiab] OR rats[Tiab] OR rat[Tiab] OR murinae[Tiab] OR muridae[Tiab] OR cottonrat[tiab] OR cottonrats[tiab] OR hamster[tiab] OR hamsters[tiab] OR cricetinae[tiab] OR rodentia[Tiab] OR rodent[Tiab] OR rodents[Tiab] OR pigs[Tiab] OR pig[Tiab] OR swine[tiab] OR swines[tiab] OR piglets[tiab] OR piglet[tiab] OR boar[tiab] OR boars[tiab] OR "sus scrofa"[tiab] OR ferrets[tiab] OR ferret[tiab] OR polecat[tiab] OR polecats[tiab] OR "mustela putorius"[tiab] OR "guinea pigs"[Tiab] OR "guinea pig"[Tiab] OR cavia[Tiab] OR callithrix[Tiab] OR marmoset[Tiab] OR marmosets[Tiab] OR cebuella[Tiab] OR hapale[Tiab] OR octodon[Tiab] OR chinchilla[Tiab] OR chinchillas[Tiab] OR gerbillinae[Tiab] OR gerbil[Tiab] OR gerbils[Tiab] OR jird[Tiab] OR jirds[Tiab] OR merione[Tiab] OR meriones[Tiab] OR rabbits[Tiab] OR rabbit[Tiab] OR hares[Tiab] OR hare[Tiab] OR diptera[Tiab] OR flies[Tiab] OR fly[Tiab] OR dipteral[Tiab] OR drosphila[Tiab] OR drosophilidae[Tiab] OR cats[Tiab] OR cat[Tiab] OR carus[Tiab] OR felis[Tiab] OR nematoda[Tiab] OR nematode[Tiab] OR nematoda[Tiab] OR nematode[Tiab] OR nematodes[Tiab] OR sipunculida[Tiab] OR dogs[Tiab] OR dog[Tiab] OR canine[Tiab] OR canines[Tiab] OR canis[Tiab] OR sheep[Tiab] OR sheeps[Tiab] OR mouflon[Tiab] OR mouflons[Tiab] OR ovis[Tiab] OR goats[Tiab] OR goat[Tiab] OR capra[Tiab] OR capras[Tiab] OR rupicapra[Tiab] OR chamois[Tiab] OR haplorhini[Tiab] OR monkey[Tiab] OR monkeys[Tiab] OR anthropoidea[Tiab] OR anthropoids[Tiab] OR saguinus[Tiab] OR tamarin[Tiab] OR tamarins[Tiab] OR leontopithecus[Tiab] OR hominidae[Tiab] OR ape[Tiab] OR apes[Tiab] OR pan[Tiab] OR paniscus[Tiab] OR "pan paniscus"[Tiab] OR bonobo[Tiab] OR bonobos[Tiab] OR troglodytes[Tiab] OR "pan troglodytes"[Tiab] OR gibbon[Tiab] OR gibbons[Tiab] OR siamang[Tiab] OR siamangs[Tiab] OR nomascus[Tiab] OR symphalangus[Tiab] OR chimpanzee[Tiab] OR chimpanzees[Tiab] OR prosimians[Tiab] OR "bush baby"[Tiab] OR prosimian[Tiab] OR bush babies[Tiab] OR galagos[Tiab] OR galago[Tiab] OR pongidae[Tiab] OR gorilla[Tiab] OR gorillas[Tiab] OR pongo[Tiab] OR pygmaeus[Tiab] OR "pongo pygmaeus"[Tiab] OR orangutans[Tiab] OR pygmaeus[Tiab] OR lemur[Tiab] OR lemurs[Tiab] OR lemuridae[Tiab] OR horse[Tiab] OR horses[Tiab] OR pongo[Tiab] OR equus[Tiab] OR cow[Tiab] OR calf[Tiab] OR bull[Tiab] OR chicken[Tiab] OR chickens[Tiab] OR gallus[Tiab] OR quail[Tiab] OR bird[Tiab] OR birds[Tiab] OR quails[Tiab] OR poultry[Tiab] OR poultries[Tiab] OR fowl[Tiab] OR fowls[Tiab] OR reptile[Tiab] OR reptilia[Tiab] OR reptiles[Tiab] OR snakes[Tiab] OR snake[Tiab] OR lizard[Tiab] OR lizards[Tiab] OR alligator[Tiab] OR alligators[Tiab] OR crocodile[Tiab] OR crocodiles[Tiab] OR turtle[Tiab] OR turtles[Tiab] OR amphibian[Tiab] OR amphibians[Tiab] OR amphibia[Tiab] OR frog[Tiab] OR frogs[Tiab] OR bombina[Tiab] OR salientia[Tiab] OR toad[Tiab] OR toads[Tiab] OR "epidalea calamita"[Tiab] OR salamander[Tiab] OR salamanders[Tiab] OR eel[Tiab] OR eels[Tiab] OR fish[Tiab] OR fishes[Tiab] OR pisces[Tiab] OR catfish[Tiab] OR catfishes[Tiab] OR siluriformes[Tiab] OR arius[Tiab] OR heteropneustes[Tiab] OR sheatfish[Tiab] OR perch[Tiab] OR perches[Tiab] OR percidae[Tiab] OR perca[Tiab] OR trout[Tiab] OR trouts[Tiab] OR char[Tiab] OR chars[Tiab] OR salvelinus[Tiab] OR "fathead minnow"[Tiab] OR minnow[Tiab] OR cyprinidae[Tiab] OR carps[Tiab] OR carp[Tiab] OR zebrafish[Tiab] OR zebrafishes[Tiab] OR goldfish[Tiab] OR goldfishes[Tiab] OR guppy[Tiab] OR guppies[Tiab] OR chub[Tiab] OR chubs[Tiab] OR tinca[Tiab] OR barbels[Tiab] OR barbus[Tiab] OR pimephales[Tiab] OR promelas[Tiab] OR "poecilia reticulata"[Tiab] OR mullet[Tiab] OR mullets[Tiab] OR seahorse[Tiab] OR seahorses[Tiab] OR mugil curema[Tiab] OR atlantic cod[Tiab] OR shark[Tiab] OR sharks[Tiab] OR catshark[Tiab] OR anguilla[Tiab] OR salmonid[Tiab] OR salmonids[Tiab] OR whitefish[Tiab] OR whitefishes[Tiab] OR salmon[Tiab] OR salmons[Tiab] OR sole[Tiab] OR solea[Tiab] OR "sea lamprey"[Tiab] OR lamprey[Tiab] OR lampreys[Tiab] OR pumpkinseed[Tiab] OR sunfish[Tiab] OR sunfishes[Tiab] OR tilapia[Tiab] OR tilapias[Tiab] OR turbot[Tiab] OR turbots[Tiab] OR flatfish[Tiab] OR flatfishes[Tiab] OR sciuridae[Tiab] OR squirrel[Tiab] OR squirrels[Tiab] OR chipmunk[Tiab] OR chipmunks[Tiab] OR suslik[Tiab] OR susliks[Tiab] OR vole[Tiab] OR voles[Tiab] OR lemming[Tiab] OR lemmings[Tiab] OR muskrat[Tiab] OR muskrats[Tiab] OR lemmus[Tiab] OR otter[Tiab] OR otters[Tiab] OR marten[Tiab] OR martens[Tiab] OR martes[Tiab] OR weasel[Tiab] OR badger[Tiab] OR badgers[Tiab] OR ermine[Tiab] OR mink[Tiab] OR minks[Tiab] OR sable[Tiab] OR sables[Tiab] OR gulo[Tiab] OR gulos[Tiab] OR wolverine[Tiab] OR wolverines[Tiab] OR minks[Tiab] OR mustela[Tiab] OR llama[Tiab] OR llamas[Tiab] OR alpaca[Tiab] OR alpacas[Tiab] OR camelid[Tiab] OR camelids[Tiab] OR guanaco[Tiab] OR guanacos[Tiab] OR chiroptera[Tiab] OR chiropteras[Tiab] OR bat[Tiab] OR bats[Tiab] OR fox[Tiab] OR foxes[Tiab] OR iguana[Tiab] OR iguanas[Tiab] OR xenopus laevis[Tiab] OR parakeet[Tiab] OR parakeets[Tiab] OR parrot[Tiab] OR parrots[Tiab] OR donkey[Tiab] OR donkeys[Tiab] OR mule[Tiab] OR mules[Tiab] OR zebra[Tiab] OR zebras[Tiab] OR shrew[Tiab] OR shrews[Tiab] OR bison[Tiab] OR bisons[Tiab] OR buffalo[Tiab] OR buffaloes[Tiab] OR deer[Tiab] OR deers[Tiab] OR bear[Tiab] OR bears[Tiab] OR panda[Tiab] OR pandas[Tiab] OR "wild hog"[Tiab] OR "wild boar"[Tiab] OR fitchew[Tiab] OR fitch[Tiab] OR beaver[Tiab] OR beavers[Tiab] OR jerboa[Tiab] OR jerboas[Tiab] OR capybara[Tiab] OR capybaras[Tiab]) NOT medline[subset])

AND

((Acepromazine[tiab] OR Acetophenazine[tiab] OR Benperidol[tiab] OR Bromperidol[tiab] OR Butaperazine[tiab] OR Carfenazine[tiab] OR Chlorproethazine[tiab] OR Chlorpromazine[tiab] OR Chlorprothixene[tiab] OR Clopenthixol[tiab] OR Cyamemazine[tiab] OR Dixyrazine[tiab] OR Droperidol[tiab] OR Fluanisone[tiab] OR Flupentixol[tiab] OR Fluphenazine[tiab] OR Fluspirilene[tiab] OR Haloperidol[tiab] OR Levomepromazine[tiab] OR Lenperone[tiab] OR Loxapine[tiab] OR Mesoridazine[tiab] OR Metitepine[tiab] OR Molindone[tiab] OR Moperone[tiab] OR Oxypertine[tiab] OR Oxyprotepine[tiab] OR Penfluridol[tiab] OR Perazine[tiab] OR Periciazine[tiab] OR Perphenazine[tiab] OR Pimozide[tiab] OR Pipamperone[tiab] OR Piperacetazine[tiab] OR Pipotiazine[tiab] OR Prochlorperazine[tiab] OR Promazine[tiab] OR Prothipendyl[tiab] OR Spiperone[tiab] OR Sulforidazine[tiab] OR Thiopropazate[tiab] OR Thioproperazine[tiab] OR Thioridazine[tiab] OR Thiothixene[tiab] OR Timiperone[tiab] OR Trifluoperazine[tiab] OR Trifluperidol[tiab] OR Triflupromazine[tiab] OR Zuclopenthixol[tiab] OR Amoxapine[tiab] OR Amisulpride[tiab] OR Aripiprazole[tiab] OR Asenapine[tiab] OR Blonanserin[tiab] OR Brexpiprazole[tiab] OR Cariprazine[tiab] OR Carpipramine[tiab] OR Clocapramine[tiab] OR Clorotepine[tiab] OR Clotiapine[tiab] OR Clozapine[tiab] OR Iloperidone[tiab] OR Levosulpiride[tiab] OR Lurasidone[tiab] OR Melperone[tiab] OR Mosapramine[tiab] OR Nemonapride[tiab] OR Olanzapine[tiab] OR Paliperidone[tiab] OR Perospirone[tiab] OR Quetiapine[tiab] OR Remoxipride[tiab] OR Reserpine[tiab] OR Risperidone[tiab] OR Sertindole[tiab] OR Sulpiride[tiab] OR Sultopride[tiab] OR Tiapride[tiab] OR Veralipride[tiab] OR Ziprasidone[tiab] OR Zotepine[tiab] OR "antipsychotic agents"[MeSH Terms] antipsychotic*[tiab] OR neuroleptic*[tiab])

AND

("substance withdrawal syndrome"[MeSH Terms] OR discontinu*[tiab] OR withdraw*[tiab] OR ceas*[tiab] OR withhold*[tiab] OR stop*[tiab] OR end[tiab] OR ended[tiab] OR ending[tiab])))

We searched PubMed, EMBASE, and Web of Science for studies until April 1, 2020 (Fig. S7). No restriction was implemented for the beginning of the searched time period. In addition, we manually searched references from included and relevant reviews. Search terms for 1) animal studies, 2) antipsychotics, and 3) withdrawal were used for filtering studies. We included antipsychotics classified in The Anatomical Therapeutic Chemical (ATC) classification system by the WHO Collaborating Centre for Drug Statistics Methodology ^10^ in the search terms. For PubMed and Web of Science we included the search filter for finding studies on animal experimentation by Hooijmans et al. (2010) ^9^ and for EMBASE we included the updated version of the EMBASE search filter for animal studies by de Vries et al. (2014) ^11^. In comparison, search terms by Hooijmans et al., 2010 and de Vries et al., 2014 were recently implemented in a publication by McCann et al., 2020 ^12^ for searching animal models of ischaemic stroke. We screened title, abstracts, and key words and implemented no language restrictions. The literature screening of title, abstract, and key words was carried out by one researcher and testing of eligibility criteria in full-text were carried out independently by two researchers. Discrepancies were resolved by consensus with an additional researcher from the review team. Authors of the original articles were contacted if information was missing in the original articles, provided contact information could be retrieved. Endnote, version X8.2 (Clarivate Analytics) was used for the literature search. We implemented the following inclusion criteria: first, only studies with behavioral assessments of VCMs in animal models after antipsychotic treatment were included. Second, only studies that reported results for at least one group treated with haloperidol, one group treated with any second generation antipsychotics ^13^, and one control group without previous antipsychotic treatment were included. Third, only studies with quantitative reports on the behavioral assessments of VCMs were included. Studies in humans, only single applications of antipsychotics (i.e. not repeated applications of antipsychotics), and studies only on first generation antipsychotics were excluded. In cases of multiple behavioral assessments at different timepoints, the first assessment after antipsychotic discontinuation was selected for the meta-analysis. A predefined spreadsheet (Microsoft Excel for Mac, version 16.12, Microsoft Corporation) was used for data collection and extraction from selected studies and data were synthesized quantitatively. We calculated standardized mean differences (SMD) and 95% confidence intervals (CIs) from outcome measures of the primary studies. If respective measures of dispersion were not available, we calculated CIs from p*-*values as recommended in the Cochrane Handbook ^14^. Data stratified according to the type of antipsychotic, effect sizes for comparisons between animals treated with antipsychotics and animals treated with placebo (i.e. control) were summarized using a forest plot. We calculated summary estimates using random-effects models, as the studies differed in several methodological aspects ^14^. Analyses were conducted according to the Cochrane Collaboration Handbook ^14^. In the main analysis, all doses per antipsychotic subgroup were included. In each study, subgroups with the same antipsychotic but different doses were combined to one subgroup to create a single pair-wise comparison (method as described in chapter 16.5.4 in the Cochrane Collaboration Handbook). In the sensitivity analysis, only the highest doses per antipsychotic subgroup in each study was included to display the effect of previous treatment with higher doses. We used Comprehensive Meta-Analysis V3 (Biostat, Engelwood, New Jersey) for the analysis.

**Cocaine self-administration.** Male and female Long Evans rats (150-350g) were implanted with subcutaneous pumps delivering haloperidol (0.5 mg/kg/d, Alzet) for 14 days. On day 14, pumps were removed and animals were fitted with intrajugular catheters under isoflurane anesthesia. 7 days after recovery from surgery, haloperidol pretreated rats and untreated controls began daily 2h cocaine self-administration sessions, where active lever presses were paired with light and tone cues for 5 seconds and cocaine delivery (0.4 mg/kg/infusion). Inactive lever presses had no consequence. Rats were maintained under mild food restriction (25 g/d) during self-administration to ensure acquisition of operant responding. After 10 days of self-administration, rats underwent extinction training (2h/d), where cues and cocaine delivery were withheld during active lever pressing. Extinction training continued for 12 days. The following day, rats were returned to the operant chamber and light/tone cue pairings were restored to the active lever, but no cocaine was delivered. Reinstatement was continued for 60m.

**Statistics.** Data were analyzed using a Student’s t-test or 1- or 2-way ANOVA with repeated measures when possible (GraphPad Software). Transformation of Ca^2+^ events into binary values was conducted using MATLAB and cluster analysis and relative data representations were performed using SAS statistics. A Pearson’s correlation coefficient was calculated to determine the relationship between Ca^2+^ events and locomotion. In all cases, statistical significance was set at p<0.05.

**References**

1. Gerfen CR, Paletzki R, Heintz N. GENSAT BAC cre-recombinase driver lines to study the functional organization of cerebral cortical and basal ganglia circuits. *Neuron* 2013; **80**(6)**:** 1368-1383.

2. Amato D, Canneva F, Cumming P, Maschauer S, Groos D, Wrosch JK *et al.* A dopaminergic mechanism of antipsychotic drug efficacy, failure, and failure reversal: the role of the dopamine transporter. *Molecular psychiatry* 2018; **25:** 2101–2118.

3. Resendez SL, Jennings JH, Ung RL, Namboodiri VM, Zhou ZC, Otis JM *et al.* Visualization of cortical, subcortical and deep brain neural circuit dynamics during naturalistic mammalian behavior with head-mounted microscopes and chronically implanted lenses. *Nat Protoc* 2016; **11**(3)**:** 566-597.

4. Marcott PF, Mamaligas AA, Ford CP. Phasic dopamine release drives rapid activation of striatal D2-receptors. *Neuron* 2014; **84**(1)**:** 164-176.

5. Marcott PF, Gong S, Donthamsetti P, Grinnell SG, Nelson MN, Newman AH *et al.* Regional Heterogeneity of D2-Receptor Signaling in the Dorsal Striatum and Nucleus Accumbens. *Neuron* 2018; **98**(3)**:** 575-587 e574.

6. Parrilla-Carrero J, Kruyer A, Chalhoub RM, Powell C, Resendez S, Amato D. Neuronal signature of an antipsychotic response. *Research Square Preprint* 2020.

7. Valjent E, Bertran-Gonzalez J, Aubier B, Greengard P, Herve D, Girault JA. Mechanisms of locomotor sensitization to drugs of abuse in a two-injection protocol. *Neuropsychopharmacology : official publication of the American College of Neuropsychopharmacology* 2010; **35**(2)**:** 401-415.

8. Rupniak NM, Jenner P, Marsden CD. Pharmacological characterisation of spontaneous or drug-associated purposeless chewing movements in rats. *Psychopharmacology* 1985; **85**(1)**:** 71-79.

9. Hooijmans CR, Tillema A, Leenaars M, Ritskes-Hoitinga M. Enhancing search efficiency by means of a search filter for finding all studies on animal experimentation in PubMed. *Lab Anim* 2010; **44**(3)**:** 170-175.

10. WHO Collaborating Centre for Drug Statistics Methodology. The Anatomical Therapeutic Chemical (ATC) classification system. *Retrieved from* [*https://wwwwhoccno*](https://wwwwhoccno).

11. de Vries RB, Hooijmans CR, Tillema A, Leenaars M, Ritskes-Hoitinga M. Updated version of the Embase search filter for animal studies. *Lab Anim* 2014; **48**(1)**:** 88.

12. McCann S, Dirnagl U, Sena ES, Bannach-Brown A, Heine K, Cruz F. Systematic review and meta-analysis of the effects of ageing on stroke outcome and treatment efficacy in animal models of ischaemic stroke. [*http://syrforguk/protocols/*](http://syrforguk/protocols/) 2020; **1–8**.

13. Leucht S, Corves C, Arbter D, Engel RR, Li C, Davis JM. Second-generation versus first-generation antipsychotic drugs for schizophrenia: a meta-analysis. *Lancet* 2009; **373**(9657)**:** 31-41.

14. Higgins. J, Green S. Cochrane Handbook for Systematic Reviews of Interventions Version 5.1.0 [updated March 2011]. *The Cochrane Collaboration Available from wwwcochrane- handbookorg* 2011.

**Supplementary Figure Legends**

**Figure S1. Haloperidol discontinuation did not alter excitatory plasticity of NAcore D1-MSNs.** Paired pulse ratio of evoked EPSCs in D1-MSNs was not altered by haloperidol discontinuation (**A**, t(16)=0.1880, p=0.8533) and D1-MSNs showed no change in AMPAr:NMDAr ratio (**B**, t(12)=0.4922, p=0.6315). The I/V relationship of NMDA currents (**C**, 2-way ANOVA Treatment F(1,111)=0.2461, p=0.6208) and NMDA current decay measured in D1-MSNs (**D**, t(16)=1.271, p=0.2219) were not changed by haloperidol discontinuation. N shown as cells/animals. Haloperidol discontinuation, HAL 14d + Abs.

**Figure S2. Ca^2+^ imaging of MSNs in the NAcore.** (**A**) Representative GCaMP6f expression and (**B**) *in vivo* signal in NAcore MSNs during locomotor testing. Representative traces of D1- (**C**) and D2-MSN (**D**) activity during recording.

**Table S1. Cells imaged *in vivo* per experimental condition.** First cocaine injection, Coca 1; Second cocaine injection, Coca 2; 14d haloperidol treatment, HAL14; Cross-sensitization, XS.

**Figure S3. Ca^2+^ events from D1- and D2-MSNs during mono- and cross-sensitization.** Ca^2+^ events in all recorded D1- or D2-MSNs during various treatment timepoints in mono-sensitized (**A**; SAL, saline injection; Coca 1, first cocaine injection; Coca 2, second cocaine injection) or cross-sensitized mice (**B**; SAL, saline injection; HAL14, 14d haloperidol treatment; XS, cross-sensitization). In the parallel plots shown in (**A**-**B**), each line represents a cell, time 0 coincides with delivery of saline or cocaine. HAL14 received no injection. K-means clustering was used to identify cellular response patterns over time during mono- (**C**) and cross-sensitization (**D**). (**C**, **D**) Biplots show points representing Ca^2+^ events/cell grouped into clusters (colored spheres) in two principal components on a correlation matrix. Size of circles in the center of each cluster is proportional to the number of cells included in the cluster. The shaded region for each cluster indicates 95% confidence and the cluster center shows the group mean. Cellular responses for each cluster are shown in (**E**, mono-sensitization) and (**F**, cross-sensitization). In (**E**), cluster 1 (222 cells) was categorized as inactivated and cluster 2 (112 cells) was categorized as unchanged. In (**F**), clusters 2 (39 cells), 3 (61 cells), 4 (5 cells), and 7 (2 cells) were categorized as activated, clusters 5 (72 cells) and 6 (26 cells) were categorized as inactivated, and cluster 1 (101 cells) was categorized as unchanged. This strategy revealed unique populations of D1- and D2-MSNs that were activated by cocaine in cross-sensitized animals. N=5 mice/grp (D2-MSNs) or 3-5 mice/grp (D1-MSNs).

**Figure S4.** **Basal activity of activated and inactivated D1- and D2-MSNs was negatively correlated with their response to cocaine.** Data represent averages of Ca^2+^ events in 5-min bins, 15-min before and after cocaine injection.

**Figure S5. G_i_-DREADD expression and function in NAcore D2-MSNs.** (**A**) Representative expression of hM4Di-mCherry in D2-MSNs in the NAcore. Number of action potentials (**B**) and representative traces (**C**) from D2-MSNs during current injections (300 pA) before (Baseline) and 5 minutes after application of CNO (CNO, 10 μM). (**D**) Cre immunolabeling was used to confirm cre-dependent G_i_-DREADD expression in the NAcore of D2-cre mice.

**Figure S6. VCMs were not present in rats after discontinuation of treatment with clinically-relevant doses of haloperidol.** VCMs were compared during 2-min of observation following a 5-min habituation period. Rats undergoing continuous treatment with haloperidol exhibited increased VCMs (Hal 14d) compared with untreated control animals (1-way ANOVA F(2,21)=79.14, p<0.0001, ****p<0.0001 vs. Control using Dunnett’s test), but VCMs were absent in rats 7d after treatment discontinuation (p=0.1236 vs. Control using Dunnett’s test). N=8 rats/grp. Haloperidol 14 days, HAL 14d; Haloperidol discontinuation, HAL 14d + Abs.

**Figure S7. PRISMA flow diagram for meta-analysis.**

**Figure S8. Motor side effects were increased after discontinuation from prolonged haloperidol treatment in animal models.** Forest plots show the standardized mean differences (SMD) and 95% confidence interval for each antipsychotic treatment subgroup compared with a control group. In (**A**), all doses per antipsychotic subgroup are included. In each study, subgroups with the same antipsychotic, but different doses are combined into one group according to the recommendations by the Cochrane Collaboration Handbook. In (**B**), sensitivity analysis shows only the highest doses per antipsychotic subgroup.

**Figure S9. Haloperidol discontinuation did not impact cocaine intake and behavioral deficits after haloperidol pretreatment were not different between males and females.** (**A**) Inactive lever pressing was not impacted by haloperidol pretreatment (2-way ANOVA Time x Treatment F(21,428)=1.232, p=0.2189) and cocaine intake did not differ between haloperidol pretreated rats and controls (inset, 2-way ANOVA Treatment F(1,21)=0.077, p=0.7835). Active lever pressing was similarly elevated throughout the first extinction session (**B**, 2-way ANOVA Treatment F(1,21)=5.437, p=0.0297) and over the course of cued reinstatement (**C**, 2-way ANOVA Treatment F(1,22)=3.379, p=0.0796) in haloperidol-pretreated rats. (**D**) Reinstated lever pressing was elevated in haloperidol pretreated animals after subtraction of the extinction baseline (i.e. active lever pressing during the first 60-min of the final extinction session 24-hour prior; t(21)=1.959 *p=0.0318). No differences in active lever pressing (**E**, 2-way ANOVA Sex F(1,154)=2.243, p=0.1363) or number of cocaine infusions (**F**, 2-way ANOVA Sex F(1,70)=2.405, p=0.1254) were observed between haloperidol pretreated male and female rats during self-administration or extinction training, although male rats in the control group took more infusions of cocaine than female control rats (2-way ANOVA Sex F(1,120)=27.78, ****p<0.0001). (**G**) No sex differences in reinstated lever pressing were observed in either treatment group (2-way ANOVA Sex F(1,19)=0.2072 p=0.6541). Animal N shown in (**A**, legend) and as scatter in (**G**).

**Figure S10. Average MSN responses during mono- and cross-sensitization.** (**A**) Average D1- and D2-MSN activity during mono-sensitization. Neither D1- (**B**) nor D2-MSN (**C**) activity correlated with locomotion in mono-sensitized mice. (**D**) When analyzed as group averages, D1-MSNs were activated and D2-MSNs were inhibited during cross-sensitization (2-way ANOVA Time x Cell Type F(8,2424)=38.68, p<0.0001). Average D1-MSN activity correlated positively with locomotion during cross-sensitization (**E**), while D2-MSN activity correlated negatively (**F**). In (**B**-**C**, **E**-**F**) data represent averages of Ca^2+^ events and locomotion across animals in 5-min bins before and after cocaine delivery. N=5 mice/grp (D2-MSNs) or 3-5 mice/grp (D1-MSNs).
