# Supplementary figures and images for: Accumbens D2-MSN hyperactivity drives antipsychotic-induced behavioral supersensitivity
Source: Mol Psychiatry. 2021 Aug 4;26(11):6159–69. doi: 10.1038/s41380-021-01235-6 (PMC8760070; doi:10.1038/s41380-021-01235-6)

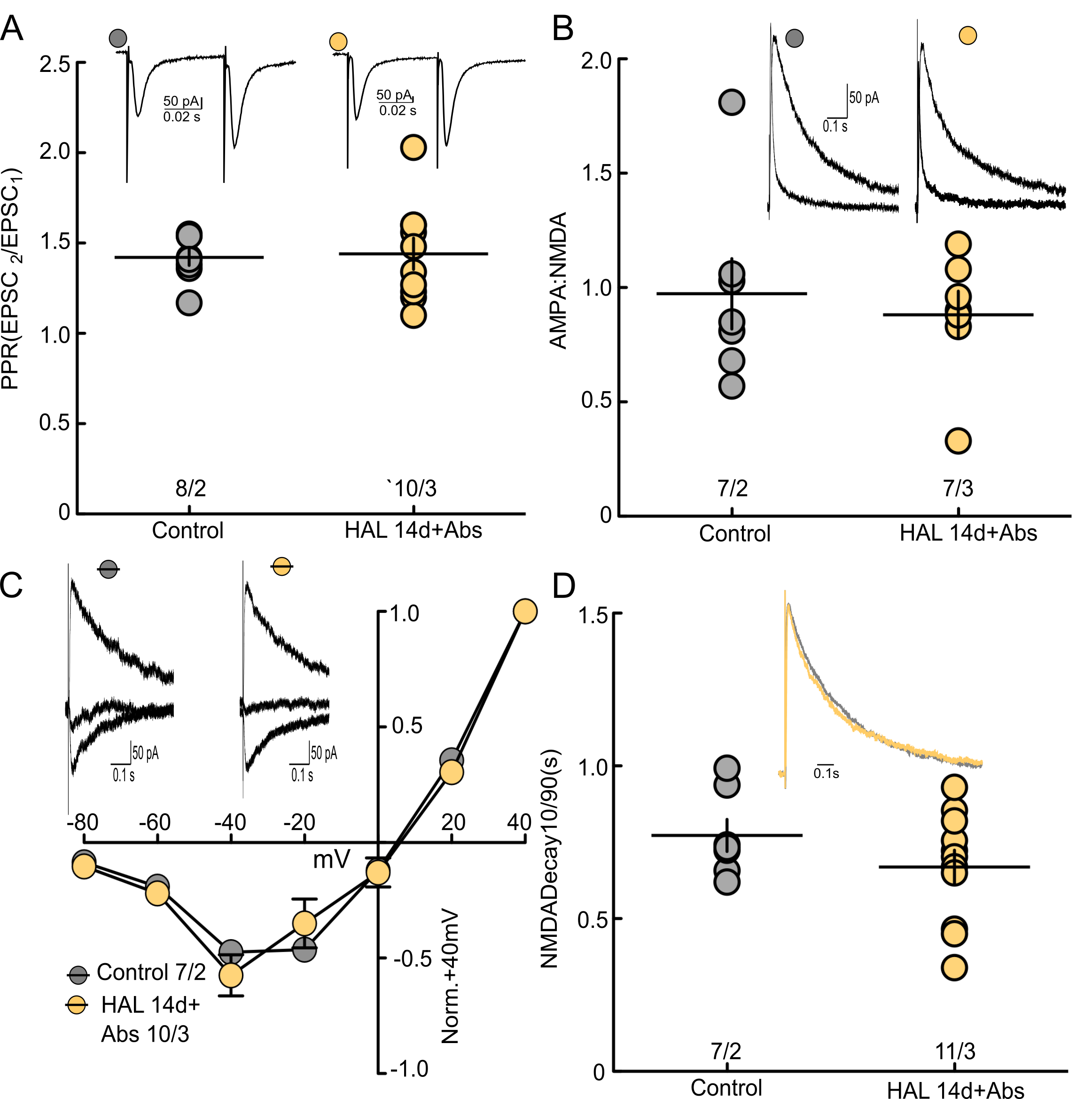

Supplement: Supplementary file 2 — Supplementary Figure 1 [file 41380_2021_1235_MOESM2_ESM.png]

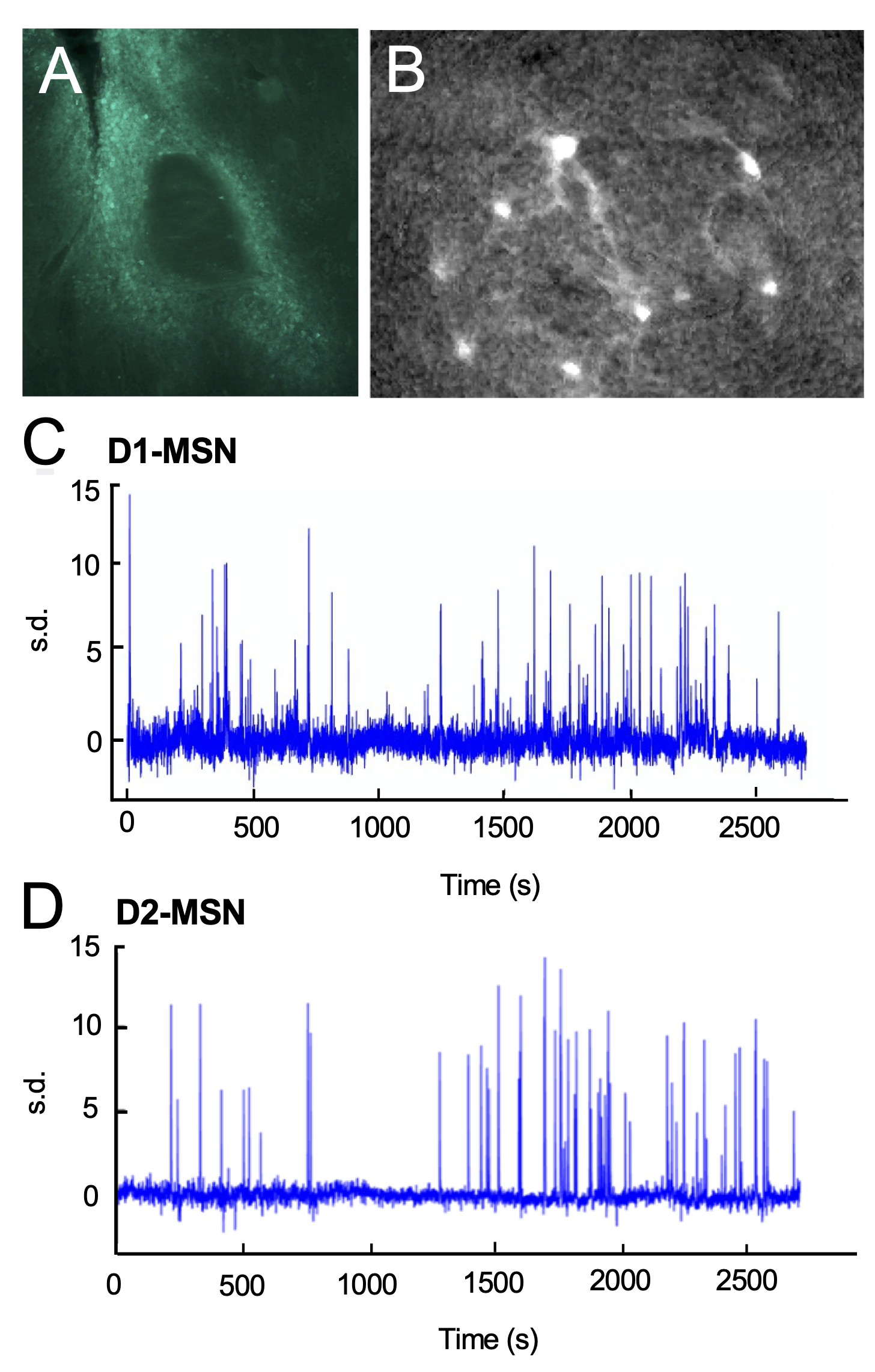

Supplement: Supplementary file 3 — Supplementary Figure 2 [file 41380_2021_1235_MOESM3_ESM.tif]

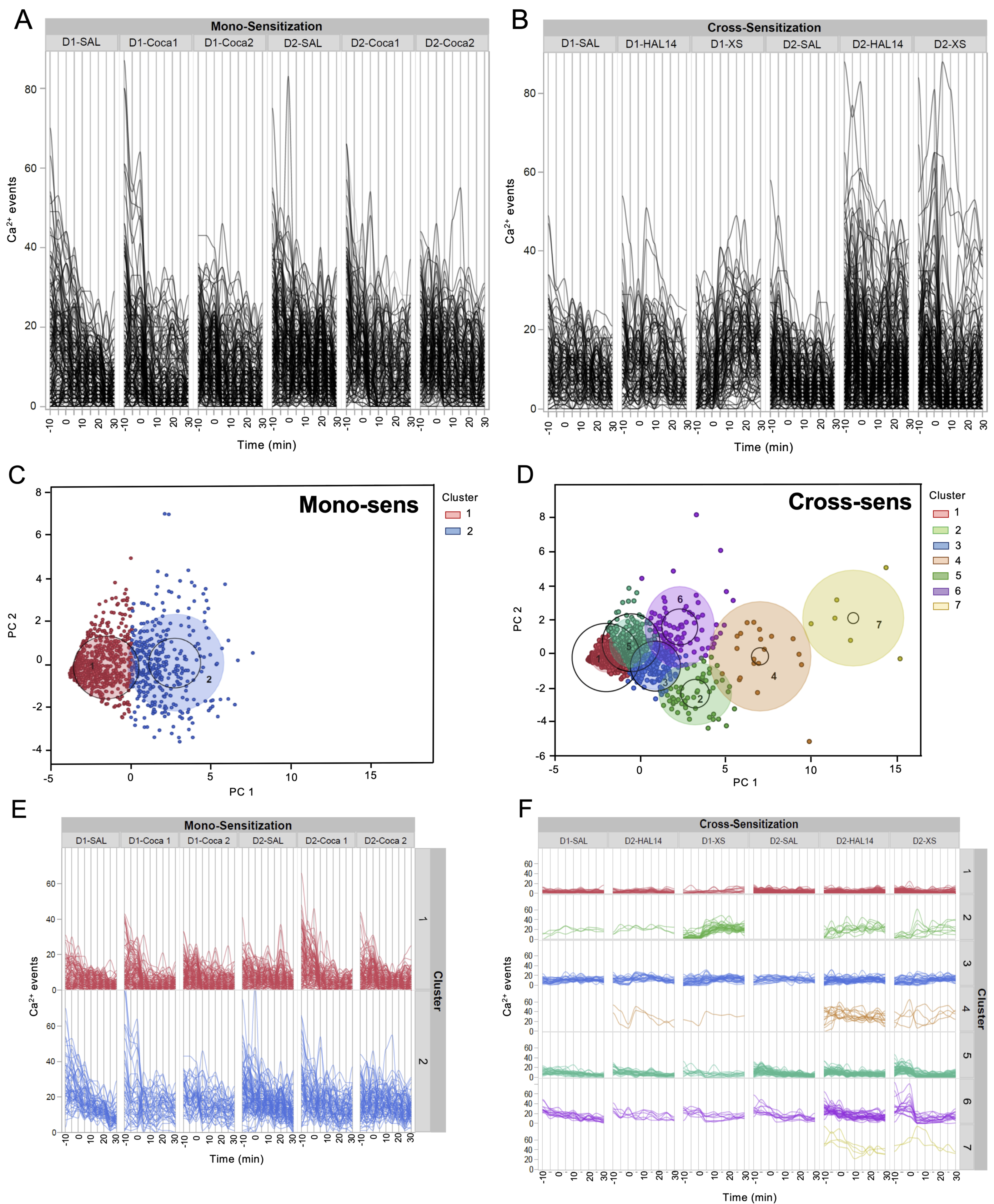

Supplement: Supplementary file 4 — Supplementary Figure 3 [file 41380_2021_1235_MOESM4_ESM.png]

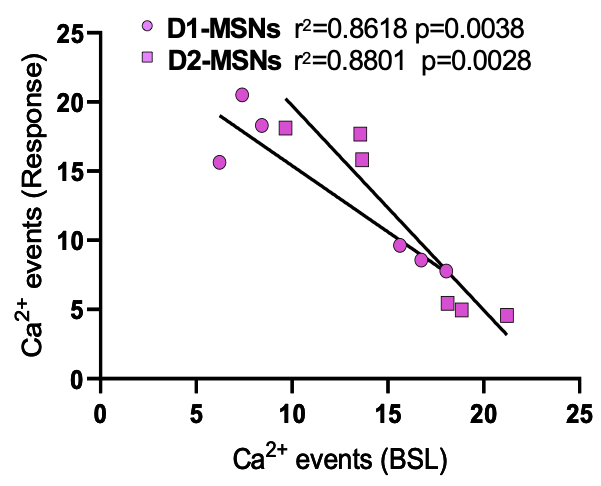

Supplement: Supplementary file 5 — Supplementary Figure 4 [file 41380_2021_1235_MOESM5_ESM.tif]

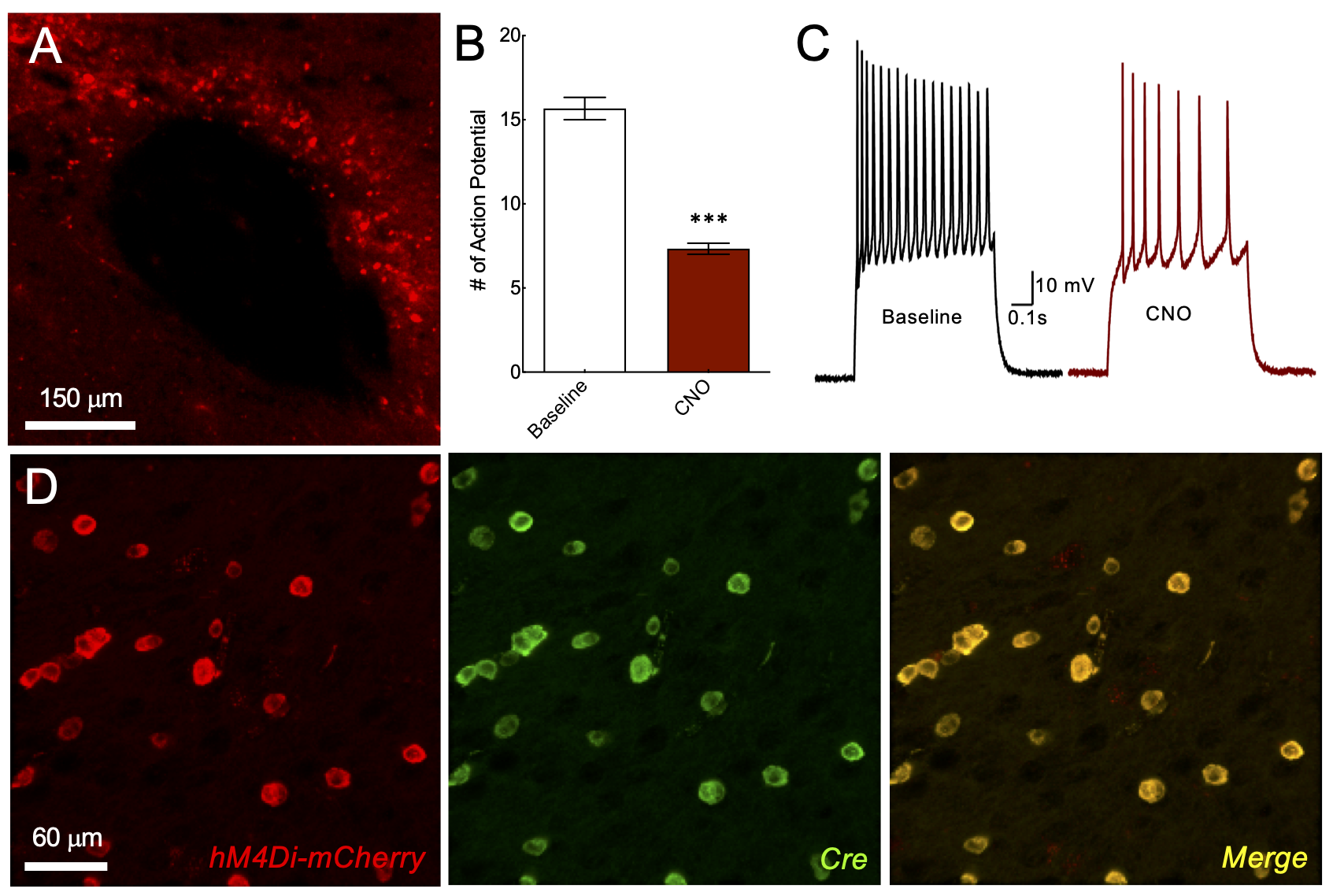

Supplement: Supplementary file 6 — Supplementary Figure 5 [file 41380_2021_1235_MOESM6_ESM.tif]

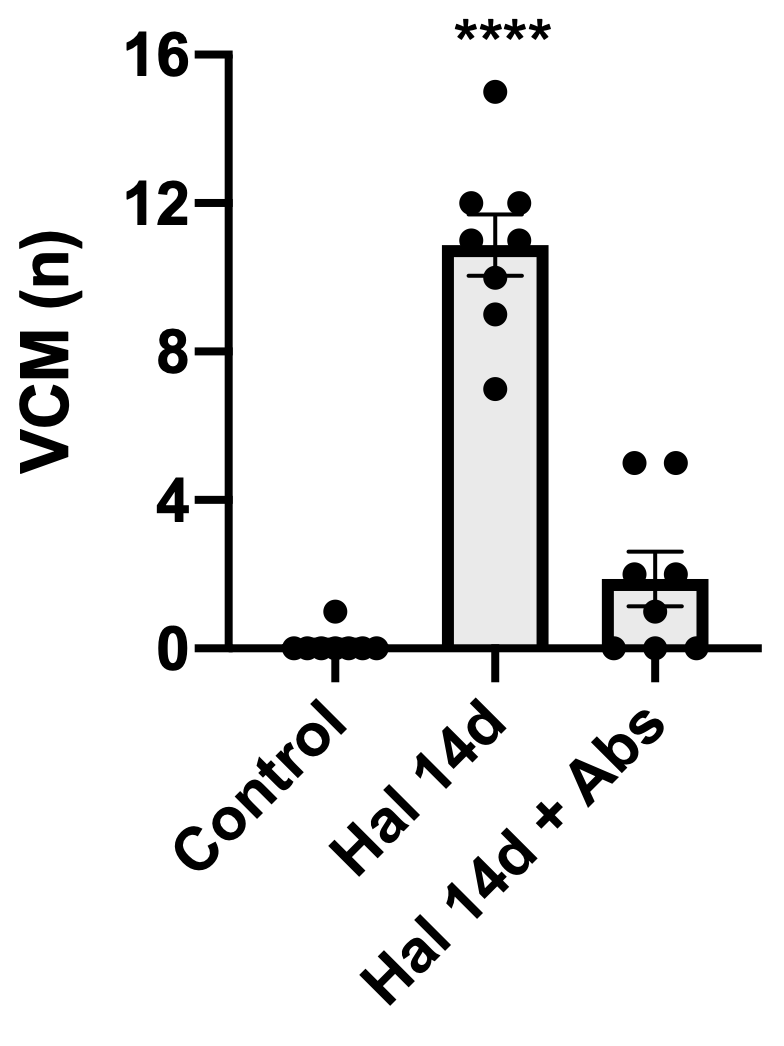

Supplement: Supplementary file 7 — Supplementary Figure 6 [file 41380_2021_1235_MOESM7_ESM.png]

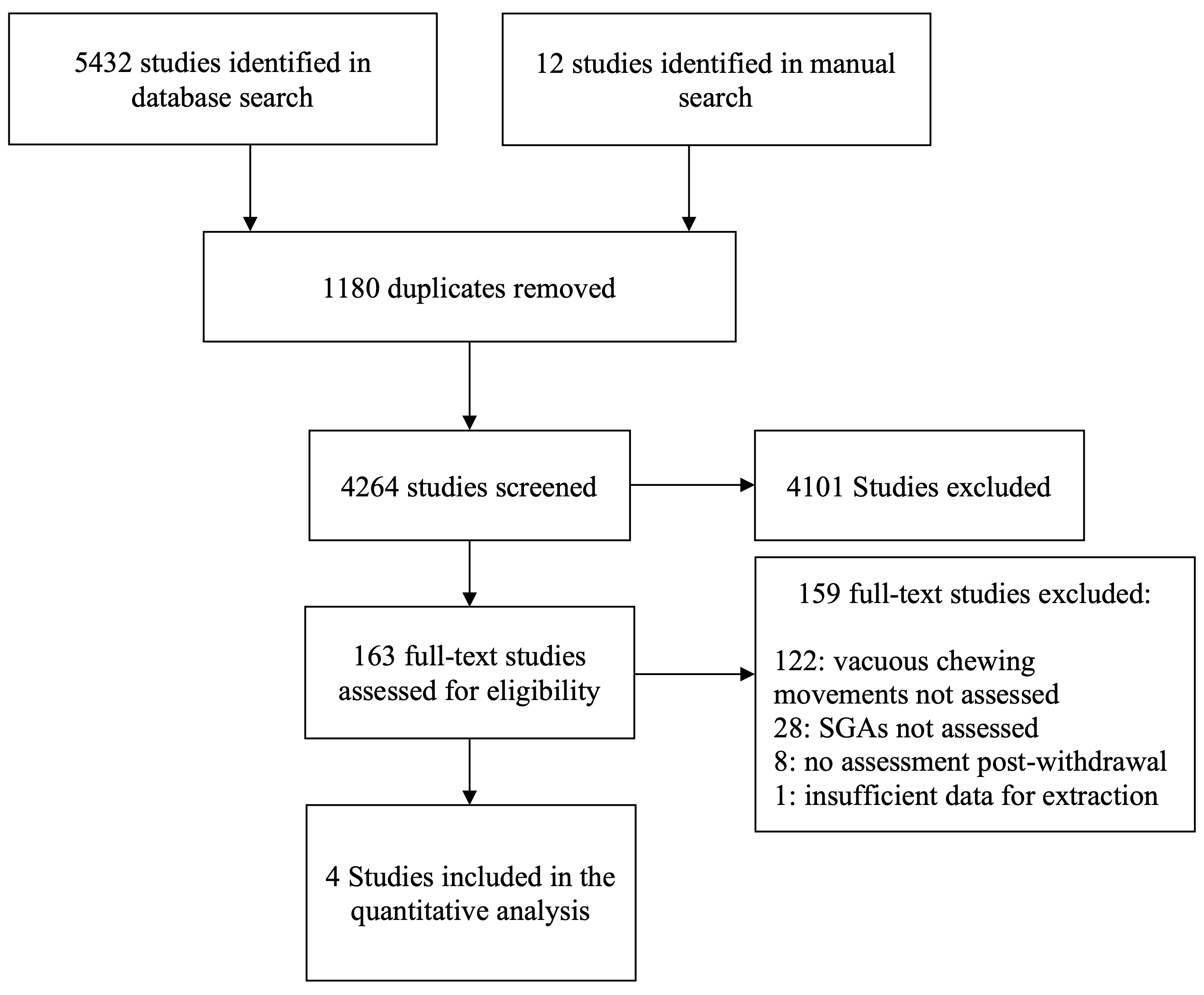

Supplement: Supplementary file 8 — Supplementary Figure 7 [file 41380_2021_1235_MOESM8_ESM.tif]

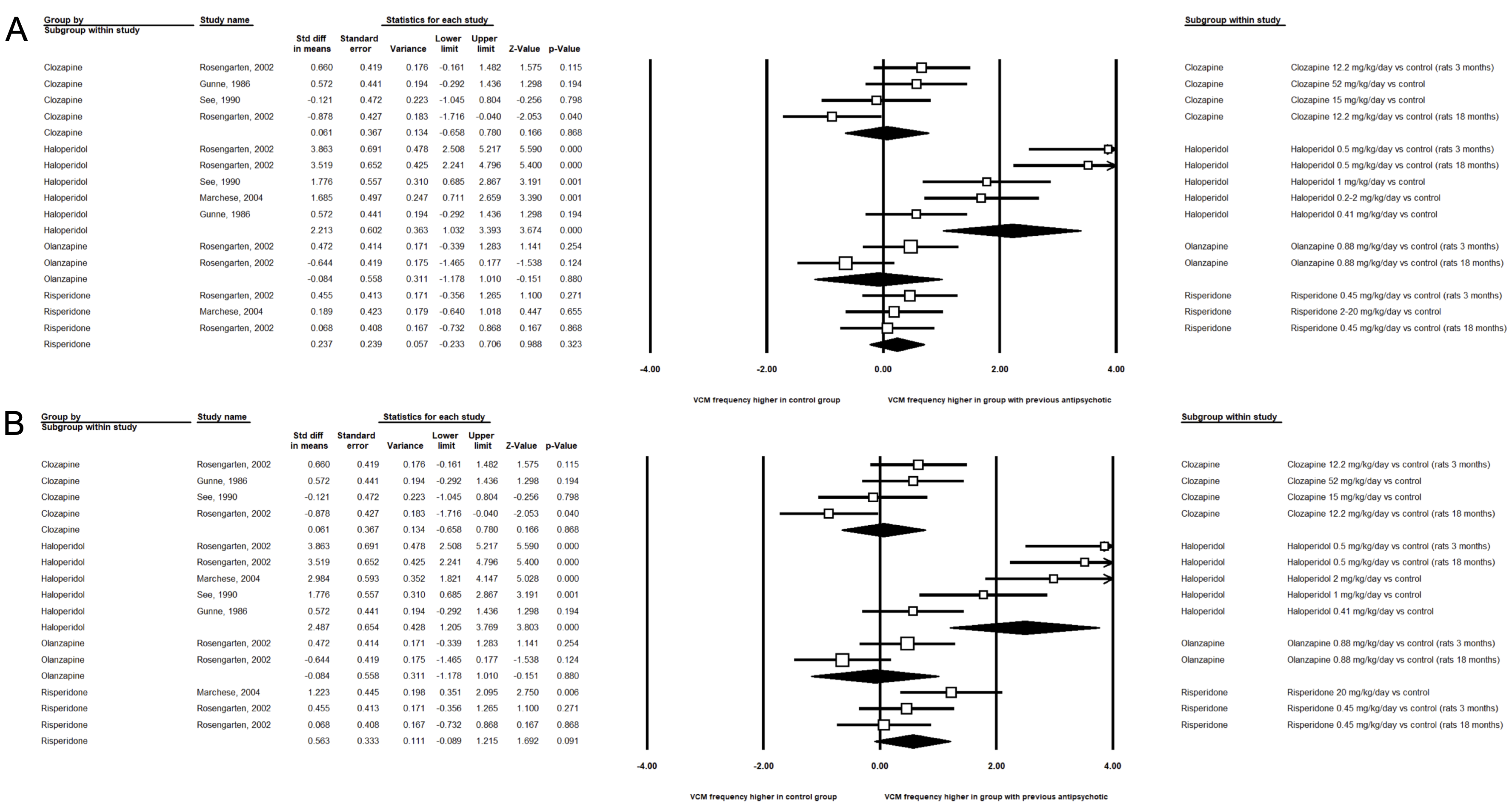

Supplement: Supplementary file 9 — Supplementary Figure 8 [file 41380_2021_1235_MOESM9_ESM.tif]

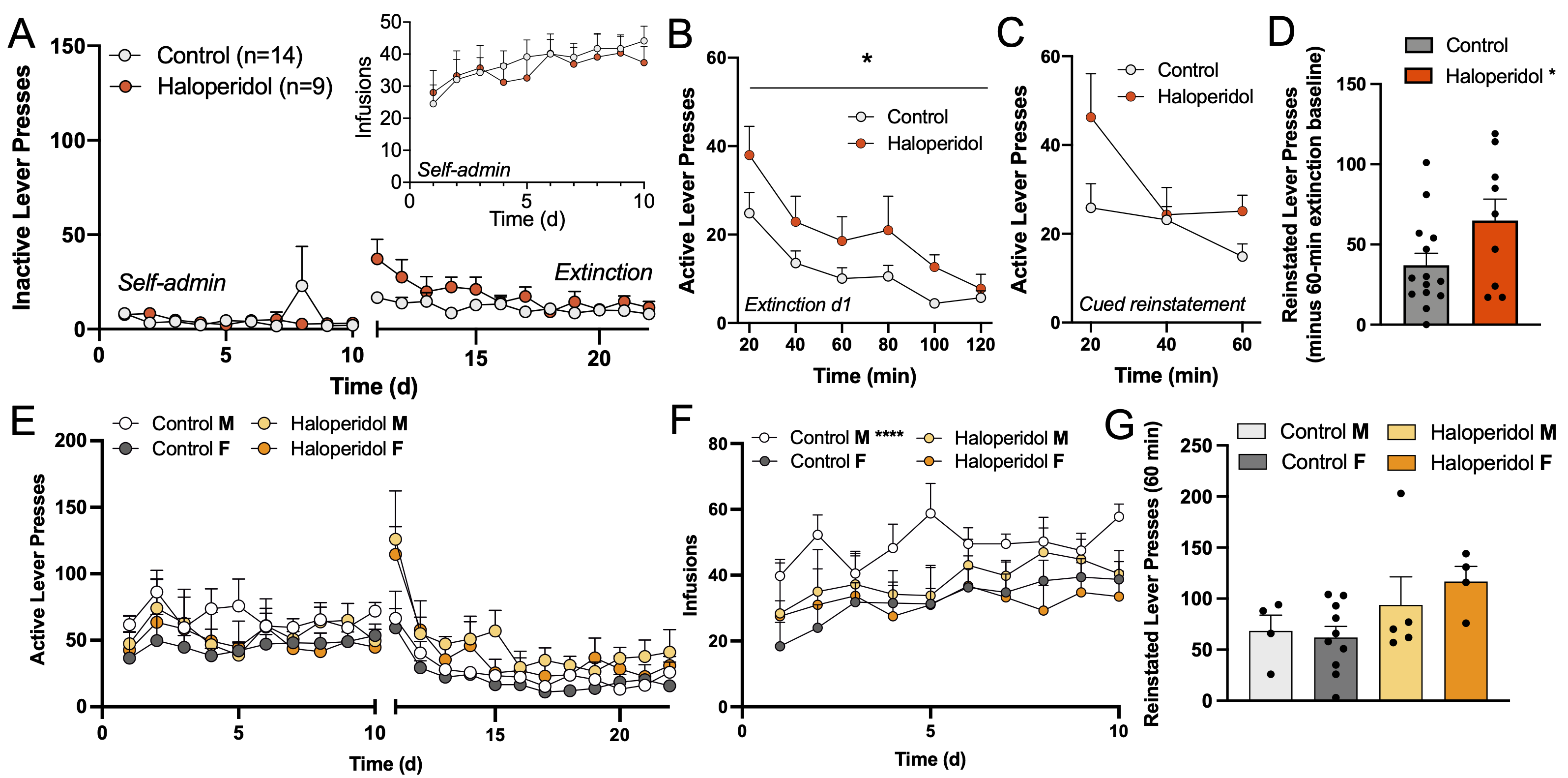

Supplement: Supplementary file 10 — Supplementary Figure 9 [file 41380_2021_1235_MOESM10_ESM.tif]

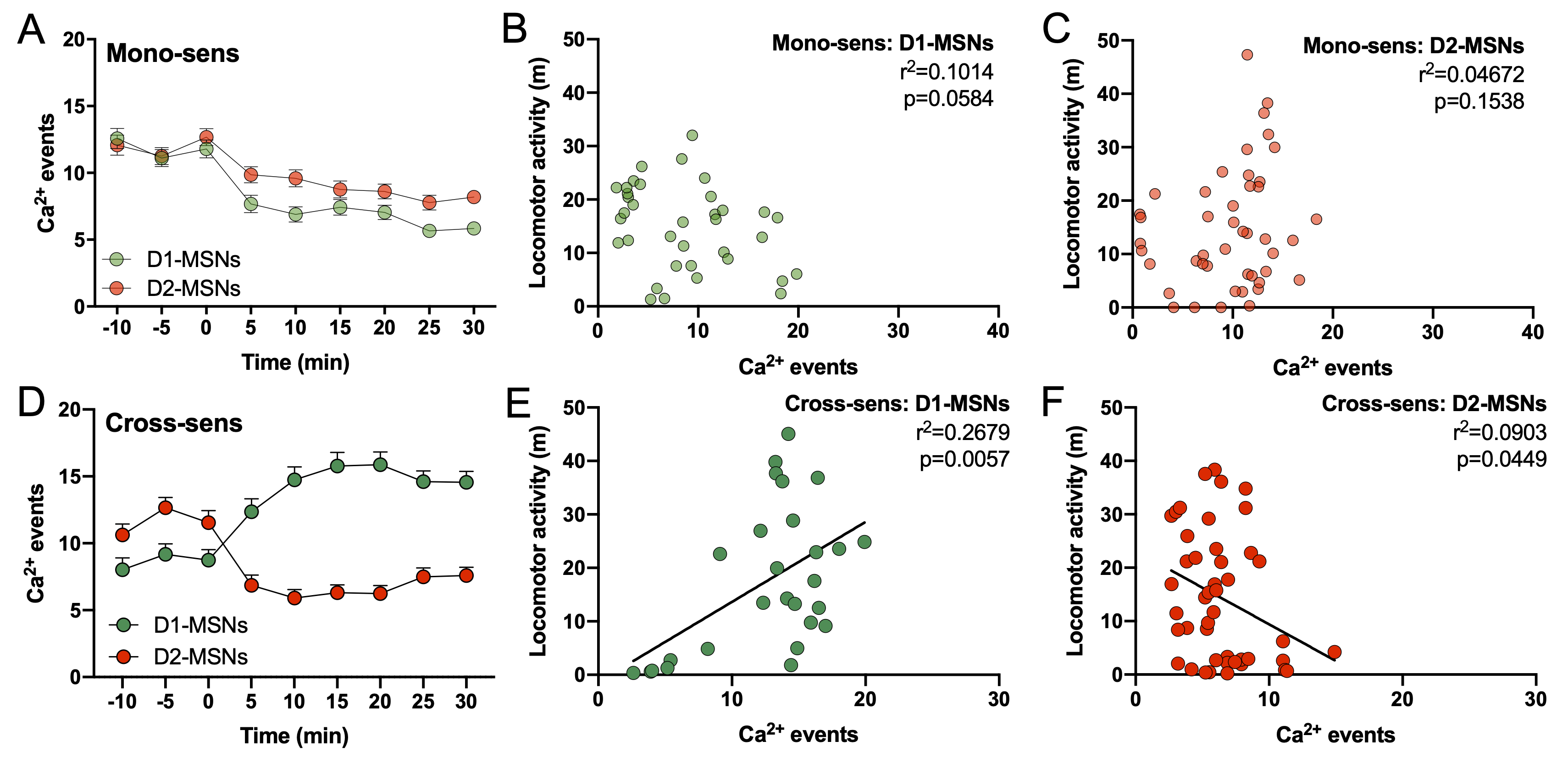

Supplement: Supplementary file 11 — Supplementary Figure 10 [file 41380_2021_1235_MOESM11_ESM.tif]

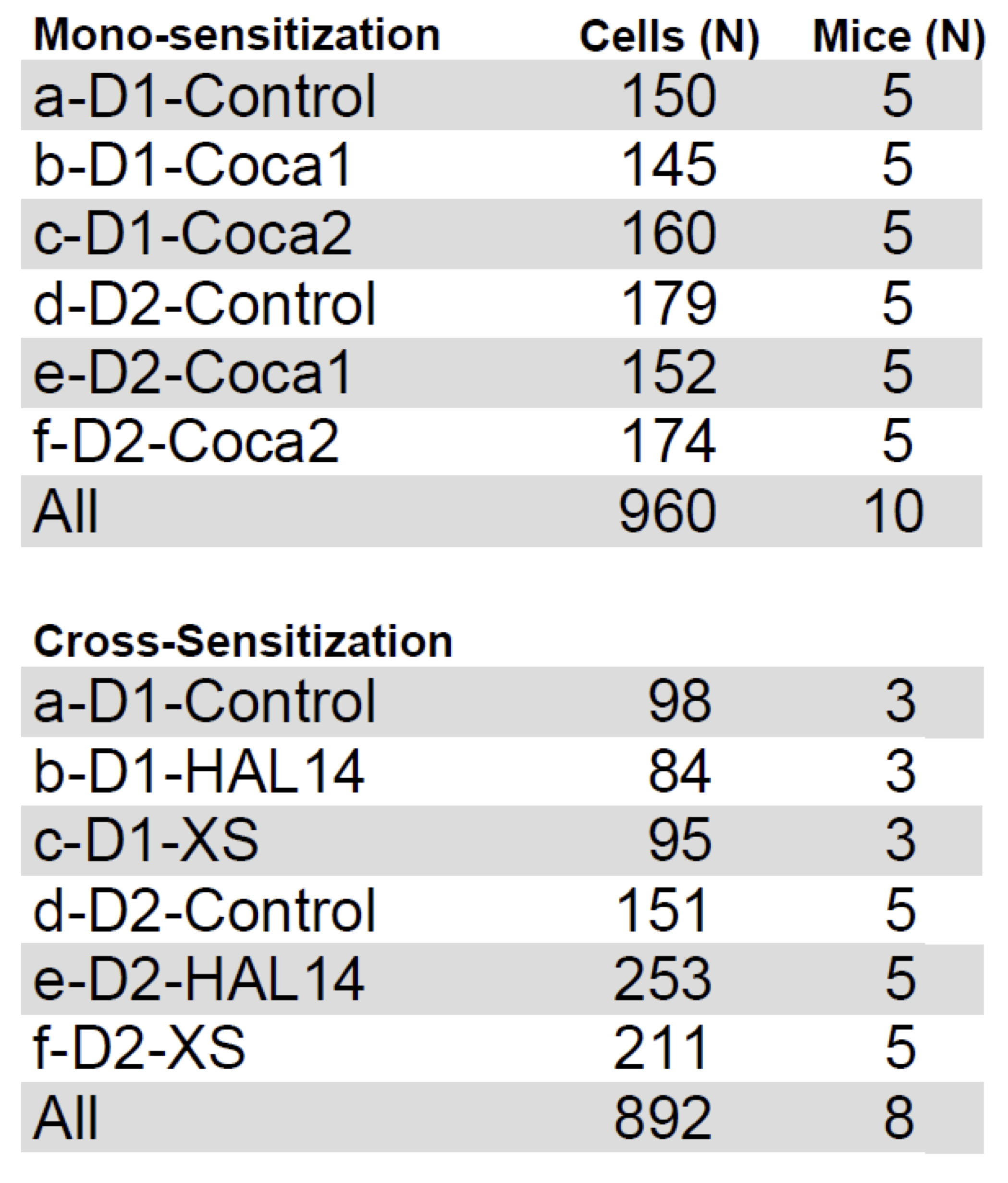

Supplement: Supplementary file 12 — Supplementary Table 1 [file 41380_2021_1235_MOESM12_ESM.png]
